# A sensitivity study on carbon nanotubes significance in Darcy–Forchheimer flow towards a rotating disk by response surface methodology

**DOI:** 10.1038/s41598-021-87956-8

**Published:** 2021-04-23

**Authors:** Anum Shafiq, Tabassum Naz Sindhu, Qasem M. Al-Mdallal

**Affiliations:** 1 School of Mathematics and Statistics, Nanjing University of Information Science and Technology, Nanjing, 210044 China; 2 Department of Statistics, Quaid-i-Azam University, 45320, Islamabad, 44000 Pakistan; 3 Department of Sciences and Humanities, FAST - National University, Islamabad, Pakistan; 4 Department of Mathematical Sciences, UAE University, P.O. Box 15551 Al-Ain, United Arab Emirates

**Keywords:** Nanoscience and technology, Mathematics and computing, Applied mathematics

## Abstract

The current research explores incremental effect of thermal radiation on heat transfer improvement corresponds to Darcy–Forchheimer (DF) flow of carbon nanotubes along a stretched rotating surface using RSM. Casson carbon nanotubes’ constructed model in boundary layer flow is being investigated with implications of both single-walled CNTs and multi-walled CNTs. Water and Ethylene glycol are considered a basic fluid. The heat transfer rate is scrutinized via convective condition. Outcomes are observed and evaluated for both SWCNTs and MWCNTs. The Runge–Kutta Fehlberg technique of shooting is utilized to numerically solve transformed nonlinear ordinary differential system. The output parameters of interest are presumed to depend on governing input variables. In addition, sensitivity study is incorporated. It is noted that sensitivity of SFC via SWCNT-Water becomes higher by increasing values of permeability number. Additionaly, sensitivity of SFC via SWCNT-water towards the permeability number is higher than the solid volume fraction for medium and higher permeability levels. It is also noted that sensitivity of SFC (SWCNT-Ethylene-glycol) towards volume fraction is higher for increasing permeability as well as inertia coefficient. Additionally, the sensitivity of LNN towards the Solid volume fraction is higher than the radiation and Biot number for all levels of Biot number. The findings will provide initial direction for future device manufacturing.

## Introduction

There are vast spectrum of uses of flow and heat transport towards a stretched surface in several engineering procedures, like wire drawing, polymer extrusion, glass fiber production, continuous casting, food and paper manufacturing, plastic film’s stretching etc. Throughout the production of such surfaces, the melting concerns from a slit and is then stretched to attain required thickness. The final product with required properties depends solely on stretching rate, procedure of stretching, and rate of cooling in process. However, due to various uses of nanoliquids flow, it has fascinated several investigators, including nanoliquid adhesive: vehicle cooling, transformer cooling, electronics cooling, electronic devices cooling and super efficient and tiny computers cooling; medical uses: safer surgery and cancer therapy via cooling and processing industries; chemicals and materials: detergency, oil and gas, drink and food, paper, printing and textiles. Many industrial technologies need intense highly efficient cooling^1–3^.

The traditional heat transport liquids, like ethylene glycol, water and thermic fluids, are commonly utilized in several industrial purposes like air-conditioning and refrigeration, transportation, microelectronics and solar thermal. Nonetheless, the restrictions in the performance of such heat transport liquids necessitate new techniques for further improving thermal transport characteristics to enhance system’s energy efficiency. It is well recognized that suspension of micro solid fragments in base liquid provides excellent potential for intensified heat transfer^4^. Nevertheless, the size of fragments in suspension contributes to precipitation, abrasion and clogging in the fluid’s flow direction. The magnificent improvements in nanotechnology have developed an novel type of heat transport liquid, known as nanofluid which has suspended fragments of size lesser than 100 nm. Nanomaterials may either be nano-powders, like *Cu*, *Al*, *CuO* and *SiC*, or CNTs. Thermal conductivity of heat transfer fluid has a significant impact on enhancing rate of heat transfer and many investigations have been delineated on thermal conductivity of nanoliquids, specifically water and ethylene glycol based nanoliquids. The experimental studies of nanoliquid thermal conductivity showed a significant increase in comparison with the base fluid. Lee et al.^5^ determined thermal conductivity of various oxide nanoliquids ($Al_{2}O_{3}$ in ethylene glycol, $Al_{2}O_{3}$ in $\hbox {H}_{2}O$, *CuO* in EG, and *CuO* in $\hbox {H}_{2}O$), and showed an increment of more than $20\%$ in *CuO*—ethylene glycol nanoliquid. But, improvement occurred when 40% increment of thermal conductivity in ethylene glycol—Cu nanoliquids recorded in^6^. Xie et al.^7^ has experimentally studied dependence of thermal conductivity of nanofluid on base liquid with various base fluids. The thermal conductivity ratio has been shown to decrease with increased thermal conductivity of base fluid. Therefore, nanoliquids can compose a fascinating option for advanced usages in heat transport in future, particularly those in micro-scale. Recent achievements concerning nanofluids for further evaluation are in Refs.^8–18^.

Porous media flows are very common among mathematicians, engineers, and modelers because of their role in geothermal energy resource, crude oil processing, oil reservoir modeling in isolation processes, water movement in reservoirs, groundwater systems etc. Flow in porous media because of heat transport becomes even more significant in procedures of thermal insulation materials, receivers and solar collectors, nuclear waste disposal, energy storage devices etc^19–21^. The existing literature experiences so much emphasis has been paid to certain porous media issues which are developed and produced using theory of the classical Darcy. Classical Darcy principle is true under lower velocity and smaller porosity circumstances. Darcy ’s rule is inadequate when there can be inertial and boundary impacts at a higher flow rate. On the other end, Reynold’s number exceeding from unity leads to non-linear flowing. In certain circumstances, the consequences of inertia and limits can not be overlooked. The impacts of inertia and boundary can’t be ignored under these circumstances. Forchheimer^22^ incorporated a square velocity expression to Darcian velocity term to estimate inertia and boundary effects. Muskat^23^ referred to this term as “Forchheimer term” that always holds for high Reynolds number. In fact, higher velocities of filtration in the momentum expression create quadratic drag for porous material. Seddeek^24^ investigated the effects of viscous dissipation and thermophoresis in DF mixed convective flow saturated porous medium. Pal and Mondal^25^ implemented DF law to studied hydromagnetic flow of variate viscosity fluid in a porous medium. Recently Shafiq et al.^26^ analyzed the influence of convective conditions and thermal slip in 3D rotating DF nanoliquids. Latest accomplishments for further assessment relating to Darcy–Forchheimer are in Refs.^27–35^.

In comparison to traditional materials, carbon nanotubes are well-suited for practically any activity involving high strength, electrical conductivity, durability, thermal conductivity, and lightweight attributes. CNTs are currently primarily utilized as synthetic additives. CNTs are widely available as a powder, which means they are heavily tangled and agglomerated. CNTs must be untangled and uniformly distributed in the substrate in order for their unique properties to unfold. By keeping this in mind the intention of this study is to look into the significance of DF flow of Casson carbon nanotubes along a rotating disk utilizing convective boundary condition. Both types of carbon nanotubes such as SWCNT and MWCNT are taken into account. Water and Ethylene glycol are considered as basic fluid. The porous space representing the Darcy Forchheimer expression is filled by an incompressible Casson fluid. Results are observed and evaluated for both SWCNTs and MWCNTs. The method of shooting (RK-4) was then utilized to solve numerically transformed nonlinear ordinary differential system. This study is concerned with essential use of carbon nanofluids in design for industrial usages such as air conditioning and refrigeration, transportation, microelectronics and solar thermal. Furthermore, an experimental scheme (RSM)^36–45^ intimately associated to a sensitivity study to examine dependence of interest bearing output parameters on input governing parameters. Remarkably, the authors conducted a sensitivity analysis based on the SFC and LNN for both types of carbon nanotubes (SWCNT and MWCNT). This study is linked with feasible rule in future gadget development. To date, such analysis is fresh and unfulfilled for the best systematic review uncovered.

## Flow problem

**Figure 1:**
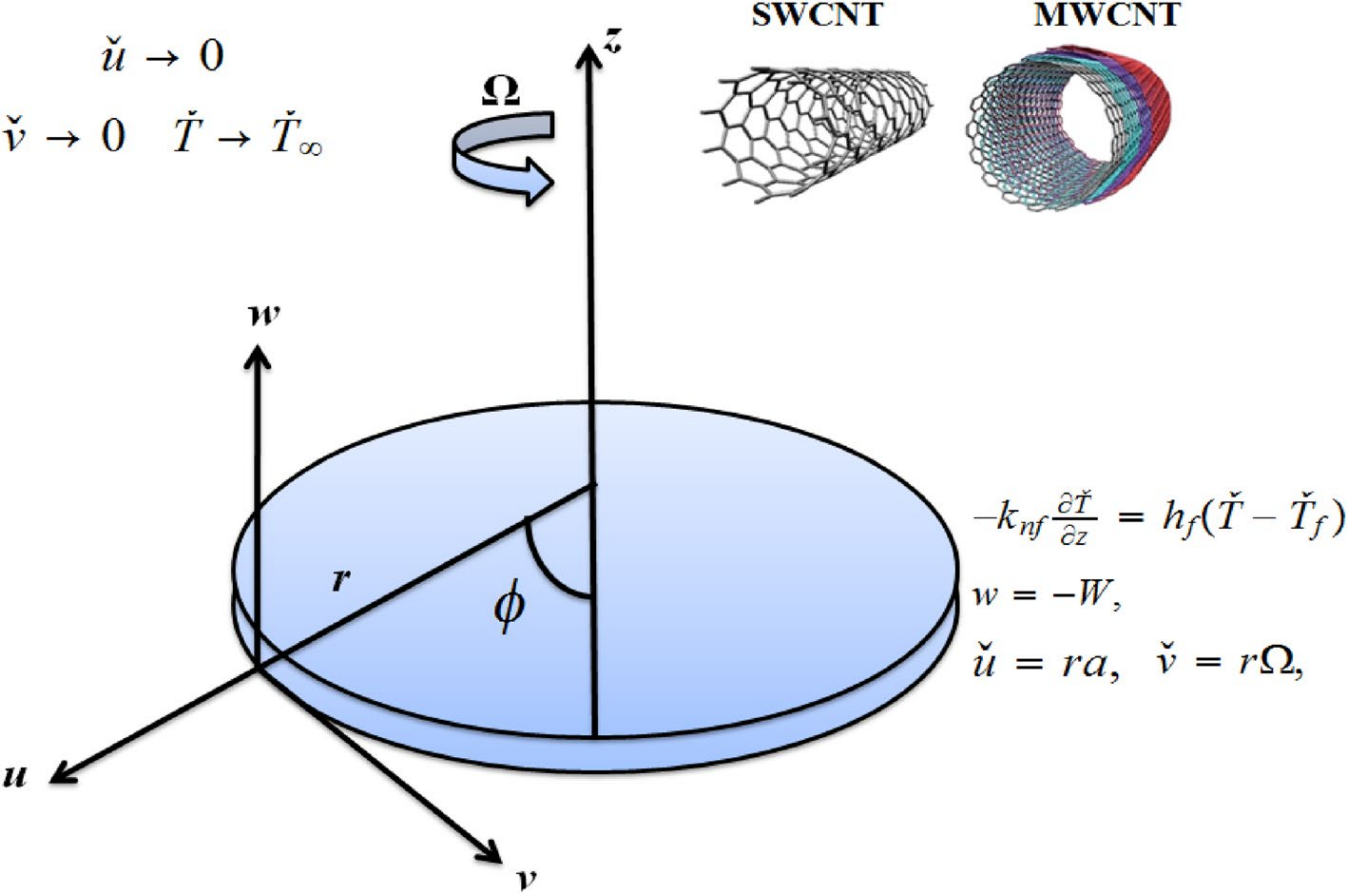
Physical systematic diagram.

A steady DF flow of Casson CNTs along a rotating disk is considered (see Fig. 1). Heat transport phenomenon is studied with subject to thermal radiation and viscous dissipation. The porous space representing the DF expression is filled by an incompressible Casson fluid. In this study, Ethylene glycol and pure water fluid are considered as base fluid and SWCNT/MWCNT is considered as nanomaterials. At $z=0$, disk spins with $\Omega$ (constant angular velocity). The consequent governing equations are^9–12,14^):

1 $$\begin{aligned} \frac{\partial \check{u}}{\partial r}+\frac{\partial \check{w}}{\partial z}=- \frac{\check{u}}{r}, \end{aligned}$$

2 $$\begin{aligned} \check{u}\frac{\partial \check{u}}{\partial r}+\check{w}\frac{\partial \check{u}}{\partial z}-\frac{\check{v}^{2}}{r}&= -\left( \rho _{nf}\right) ^{-1}\frac{\partial p}{\partial r}+\left( 1+\frac{1}{\gamma _{1}}\right) \frac{\mu _{nf}}{\rho _{nf}}\left( \frac{\partial ^{2}\check{u}}{\partial r^{2}}-\frac{\check{u}}{r^{2}}+\frac{1}{r}\frac{\partial \check{u}}{\partial r}+\frac{\partial ^{2}\check{u}}{\partial z^{2}}\right) +\frac{\left( \rho \beta \right) _{nf}}{\rho _{nf}}\check{g}\left( \check{T}-\check{T}_{\infty }\right) \nonumber \\&\quad -\frac{\mu _{nf}}{\rho _{nf}}\frac{\check{u}}{K^{*}}-F^{*}u^{2}- \frac{\sigma B_{0}^{2}}{\rho }\check{u}^{2}, \end{aligned}$$

3 $$\begin{aligned} \check{u}\frac{\partial \check{v}}{\partial r}+\frac{\check{u}\check{v}}{r}+ \check{w}\frac{\partial \check{v}}{\partial z}&= \left( 1+\frac{1}{\gamma _{1}}\right) \frac{\mu _{nf}}{\rho _{nf}}\left( \frac{\partial ^{2}\check{v} }{\partial r^{2}}+\frac{1}{r}\frac{\partial \check{v}}{\partial r}-\frac{ \check{v}}{r^{2}}+\frac{\partial ^{2}\check{v}}{\partial z^{2}}\right) + \frac{\left( \rho \beta \right) _{nf}}{\rho _{nf}}\check{g}\left( \check{T}- \check{T}_{\infty }\right) \nonumber \\&\quad -\frac{\mu _{nf}}{\rho _{nf}}\frac{\check{v}}{K^{*}}-F^{*}\check{v} ^{2}-\frac{\sigma B_{0}^{2}}{\rho }\check{v}, \end{aligned}$$

4 $$\begin{aligned} \check{u}\frac{\partial \check{w}}{\partial r}+\check{w}\frac{\partial \check{w}}{\partial z}&= -\frac{1}{\rho _{nf}}\frac{\partial p}{\partial z} +\left( 1+\frac{1}{\gamma _{1}}\right) \frac{\mu _{nf}}{\rho _{nf}}\left( \frac{\partial ^{2}\check{w}}{\partial r^{2}}+\frac{\partial ^{2}\check{w}}{ \partial z^{2}}+\frac{1}{r}\frac{\partial \check{w}}{\partial r}\right) , \end{aligned}$$

5 $$\begin{aligned} \check{w}\frac{\partial \check{T}}{\partial z}+\check{u}\frac{\partial \check{T}}{\partial r}&= \frac{1}{\left( \rho c_{p}\right) _{nf}}\left( k_{nf}+\frac{16\sigma _{1}^{*}}{3k_{1}^{*}}T^{3}\right) \left( \frac{ \partial ^{2}\check{T}}{\partial r^{2}}+\frac{1}{r}\frac{\partial \check{T}}{ \partial r}+\frac{\partial ^{2}\check{T}}{\partial z^{2}}\right) \nonumber \\&\quad +2\frac{\mu _{nf}}{\left( \rho c_{p}\right) _{nf}}\left( 1+\frac{1}{\gamma _{1}}\right) \left[ \left( \frac{\partial \check{u}}{\partial r}\right) ^{2}+ \frac{\check{u}^{2}}{r^{2}}+\left( \frac{\partial \check{w}}{\partial z} \right) \right] \nonumber \\&\quad+\frac{\mu _{nf}}{\left( \rho c_{p}\right) _{nf}}\left( 1+\frac{1}{\gamma _{1}}\right) \left[ \left( \frac{\partial \check{v}}{\partial z}\right) ^{2}+\left( \frac{\partial \check{w}}{\partial r}+\frac{\partial \check{u}}{ \partial z}\right) ^{2}\right. \nonumber \\&\quad \left. \left( r\frac{\partial }{\partial r}\left( \frac{\check{v}}{r} \right) ^{2}\right) \right] , \end{aligned}$$

with^12^:

6 $$\begin{aligned} \check{u}= & {} \, ra,{ \ }\check{v}=r\Omega ,{ \ }w=-W,\, -k_{nf} \frac{\partial \check{T}}{\partial z}=h_{f}\left( \check{T}-\check{T} _{f}\right) ,\text { at }z=0,\nonumber \\ \check{u}\rightarrow & {} 0,{ \ }\check{v}\rightarrow 0,{\, \, }\check{T} \rightarrow \check{T}_{\infty }\text { when }z\rightarrow \infty . \end{aligned}$$

An important condition is incorporated at boundary, namely convective condition. The heat transport through surface improves temperature and hence thermal conductivity of nanofluids because of convective condition. The application of convective boundary condition is therefore best adapted as a standard compared to isothermal conditions. In these situations, lower surface is heated via hot liquid that have $\check{T}_{f}$ temperature with $h_{f}$ coefficient of heat transfer. In these situations, $k_{n_{f}}$ is the nanofluid’s thermal conductivity inside the boundary layer, $u=ra$ is stretched velocity, $v=r\Omega$ is rotational speed. Suction is considered in the current boundary, adding/removing reactants, reducing the drag, cooling the surface, fluid scaling or preventing corrosion. Consequently, suction can be used with stretching/shrinking surfaces to effectively control the growth/decay of the momentum boundary layer. Suction is adapted to established boundary that adds/removes reactants, reduces drag, cools surface, prevents fluid corrosion or scaling. Consequently, suction can be used with stretching/shrinking sheets to effectively control the growth/decay of the momentum boundary layer.

The effective characteristics of carbon nanotubes are given below^13^:

7 $$\begin{aligned} {\check{\mu }}_{n_{f}}&= \frac{\mu _{_{f}}}{\left( 1-\phi \right) ^{2.5}}, \text { }{\check{\alpha }}_{nf}=\frac{\check{k}_{n_{f}}}{\left( \rho c_{p}\right) _{n_{f}}},\text { }{\check{\rho }}_{n_{f}}=\left( 1-\phi \right) \rho _{_{f}}+\phi \rho _{_{CNT}},\text { }\check{v}_{n_{f}}=\frac{{\check{\mu }} _{n_{f}}}{{\check{\rho }}_{n_{f}}}, \nonumber \\ \frac{\check{k}_{n_{f}}}{\check{k}_{_{f}}}&= \frac{\left( 1-\phi \right) +2\phi \frac{k_{_{CNT}}}{k_{_{CNT}}-k_{_{f}}}\ln \frac{k_{_{CNT}}+k_{_{f}}}{ 2k_{f}}}{\left( 1-\phi \right) +2\phi \frac{k_{_{f}}}{k_{_{CNT}}-k_{_{f}}} \ln \frac{k_{_{CNT}}+k_{_{f}}}{2k_{_{f}}}},{ \ }(\rho c_{p})_{n_{f}}=(1-\phi )(\rho c_{p})_{_{f}}+\phi (\rho c_{p})_{_{CNT}}, \nonumber \\ (\rho \beta )_{n_{f}}&= (1-\phi )(\rho \beta )_{_{f}}+\phi (\rho \beta )_{_{CNT}}, \end{aligned}$$

where CNTs solid volume fraction is $\phi ,$ CNTs thermal conductivity is $k_{_{CNT}},$ Base fluid’s thermal conductivity is $k_{{f}},$ nanofluid’s dynamic viscosity is ${\check{\mu }}_{n_{f}},$ nanofluid’s density ${\check{\rho }} _{n_{f}},$ CNTs heat capacity $(\rho c_{p})_{_{CNT}}.$ Thermophysical properties of different base liquids and CNTs are listed in Table 1.

**Table 1:** Thermophysical properties of different base liquids and CNTs.

	$\rho$/$(\text {kg}\,\text {m}^{-3})$	$c_{p}$/$(\text {J}\,\text {kg}^{-1}\,\text {K}^{-1})$	*k*/$(\text {W}\,\text {m}^{-1}\,\text {K}^{-1})$	$\beta$/$K^{-1}$	$\Pr$
**Base fluids physical properties**					
Water (W)	997.1	4,179	0.613	$21\times 10^{-5}$	6.2
Ethylene glycol (EG)	1115	2,430	0.253	$65\times 10^{-5}$	203.63
Engine oil	884	1,910	0.144	$70\times 10^{-5}$	6,450
Glycerin	1259.9	2427	0.286	$48\times 10^{-5}$	6.78

**Nanomaterial physical properties**					
Copper $\left( C_{u}\right)$	8,933	385	401	$1.67\times 10^{-5}$	−
Silver $\left( A_{g}\right)$	10,500	235	429	$1.89\times 10^{-5}$	−
Alumina $\left( Al_{2}O_{3}\right)$	3, 970	765	40	$0.85\times 10^{-5}$	−
Titanium $\left( TiO_{2}\right)$	4,250	6,862	8.9538	$0.9\times 10^{-5}$	−
SWCNT	2,600	425	6,600	$27\times 10^{-5}$	−
MWCNT	1,600	796	3,000	$44\times 10^{-5}$	−

We take transformations into consideration

8 $$\begin{aligned} \check{u}= & {} r\Omega f^{\prime }\left( \eta \right) ,\text { }\check{v} =r\Omega g\left( \eta \right) ,{ \ }\check{w}=\sqrt{2\Omega \nu _{_{f}}} f\left( \eta \right) {\, \, }\eta =\left( \frac{2\Omega }{\nu _{_{f}}} \right) ^{\frac{1}{2}}z, \nonumber \\ p= & {} p_{\infty }-\Omega \mu _{_{f}}\text { }P\left( \eta \right) ,{ \ \ } \theta =\frac{\check{T}-\check{T}_{\infty }}{\check{T}_{f}-\check{T}_{\infty }}. \end{aligned}$$

where non-dimensional distance along axis of rotation is defined as $\eta$ and *f*, *g* and $\theta$ are functions of $\eta$. Replacing the above mentioned transformations into Eqs. (1)–(6), we attain the following set of differential equations:

9 $$\begin{aligned}&\left. \frac{1}{\left( 1-\phi \right) ^{2.5}(1-\phi +\phi \frac{\rho _{_{_{CNT}}}}{\rho _{_{_{f}}}})}\left( 1+\frac{1}{\gamma _{1}}\right) \left( 2f^{\prime \prime \prime }-k_{1\text { }}f^{\prime }\right) +2ff^{\prime \prime }-f^{\prime ^{2}}+g^{2}\right. \nonumber \\&\left. -F_{r}\text { }f^{\prime ^{2}}-\frac{M_{1}^{2}}{\left( 1-\phi +\phi \frac{\rho _{_{_{CNT}}}}{\rho _{_{_{f}}}}\right) }f^{\prime }+\frac{(1-\phi + \frac{\left( \rho \beta \right) _{_{CNT}}}{\left( \rho \beta \right) _{f}} \phi )}{(1-\phi +\phi \frac{\rho _{_{_{CNT}}}}{\rho _{_{_{f}}}})}\lambda \theta =0,\right. \end{aligned}$$

10 $$\begin{aligned}&\left. \frac{1}{\left( 1-\phi \right) ^{2.5}(1-\phi +\phi \frac{\rho _{_{_{CNT}}}}{\rho _{_{_{f}}}})}\left( 1+\frac{1}{\gamma _{1}}\right) \left( g^{\prime \prime }-k_{1\text { }}g\right) +2fg^{\prime }-2f^{\prime }g\right. \nonumber \\&\left. -F_{r}g^{2}-\frac{M_{1}^{2}}{\left( 1-\phi +\phi \frac{\rho _{_{_{CNT}}}}{\rho _{_{_{f}}}}\right) }g+\frac{(1-\phi +\frac{\left( \rho \beta \right) _{_{CNT}}}{\left( \rho \beta \right) _{f}}\phi )}{(1-\phi +\phi \frac{\rho _{_{_{CNT}}}}{\rho _{_{_{f}}}})}\lambda \theta =0,\right. \end{aligned}$$

11 $$\begin{aligned}&\left. \frac{\frac{k_{nf}}{k_{f}}}{\left( 1-\phi +\phi \frac{\left( \rho C_{p}\right) _{CNT}}{\left( \rho C_{p}\right) f}\right) }\left( 1+\frac{4}{3} \frac{k_{_{f}}}{k_{n_{f}}}R_{*}\right) \theta ^{\prime \prime }+\Pr f \text { }\theta ^{\prime }+6\frac{\Pr Ec}{\left( 1-\phi \right) ^{2.5}} f^{\prime 2}\right. \nonumber \\&\left. +2\frac{\Pr Ec}{\left( 1-\phi \right) ^{2.5}}\left( 1+\frac{1}{ \gamma _{1}}\right) \left( f^{\prime \prime 2}+g^{\prime 2}\right) =0,\right. \end{aligned}$$

12 $$\begin{aligned}&\left. f(0)=S_{1},{ \ }f^{\prime }\left( 0\right) =\delta _{1},{ \ }g\left( 0\right) =1,{\, \, }\frac{k_{n_{f}}}{k_{_{f}}}\theta ^{\prime }(0)+\gamma _{2}(1-\theta (0))=0,\right. \nonumber \\&\left. f^{\prime }\left( \infty \right) \rightarrow 0,{ \ }g(\infty )\rightarrow 0,{\, \, }\theta (\infty )\rightarrow 0.\right. \end{aligned}$$

Here $\lambda ,$ $k_{1},$ $F_{r},$ $\Pr ,$ $R_{*},$ $S_{1},$ *Ec*,  $\delta _{1}$ and $\gamma _{2}$ are defined mixed convective number, permeability number, Inertia coefficient, Prandtl parameter, radiation parameter, suction parameter, Eckert number, stretching-strength parameter and Biot number respectively and describe as follows

13 $$\begin{aligned} \lambda& = \frac{g\beta _{T}\rho _{f}\left( T_{w}-T_{\infty }\right) r}{ \upsilon _{f}\Omega },{ \ }k_{1}=\frac{\upsilon _{f}}{\Omega K^{*}}, { \ }F_{r}=\frac{cd}{\sqrt{K^{*}}},{\, }\Pr =\frac{\upsilon _{f} }{\alpha _{f}},{ \ }R_{*}=\frac{4\sigma _{1}^{*}T_{\infty }^{3} }{k_{1}^{*}k_{f}}, \nonumber \\ S_{1}&= \frac{W}{\sqrt{2\Omega \upsilon _{f}}},{ \ }Ec=\frac{ r^{2}\Omega ^{2}}{\left( c_{p}\right) _{_{f}}\text { }k\left( T_{w}-T_{\infty }\right) },{ \ \ }\gamma _{2}=\frac{h_{f}}{k_{f}}\sqrt{\frac{\upsilon _{f}}{2\Omega }},{\ \ \ }\delta _{1}=\frac{a}{\Omega }. \end{aligned}$$

The physical quantities are defined in the following forms, namely LSFC and LNN

14 $$\begin{aligned} C_{fr}=\frac{2\tau _{rz}}{\rho U_{w}^{2}},{ \ \ \ \ \ \ \ }Nu_{r}=\frac{ xq_{w}}{K\left( \check{T}-\check{T}_{\infty }\right) }. \end{aligned}$$

The dimensionless forms are as

15 $$\left( \mathrm {Re}_{r}\right) ^{1/2}C_{fr}=\frac{1}{\left( 1-\phi \right) ^{2.5}}\left( 1+\frac{1}{\gamma _{1}}\right) \sqrt{\left( f^{\prime\prime }\left( 0\right) \right) ^{2}+\left( g^{\prime }\left( 0\right) \right) ^{2}} ,$$

16 $$\left( \mathrm {Re}_{r}\right) ^{-1/2}Nu_{r}=-\left( \frac{k_{n_{f}}}{k_{_{f}}}+ \frac{4}{3}R_{*}\right) \theta ^{\prime }(0).$$

## Numerical computational simulation

A numerical computational simulation which interacts with quantity interpretation is basically known as mathematical experiment. It’s a process containing of a series of data tests, using a computer program to mimic the behaviors of the real world scenario. A computational analysis is carried out to find out output result of a change in code, because of several input variables. Conclusion on importance and pertinent variables may also be concluded in the end study. The model dependence is defined using RSM (see^41,42^) in terms of relationship among input factors and output response.

In the entire investigation, there are four interest parameters and total of $``12''$ independent input parameters. However, we mainly highlighted sensitivity assessment for interest parameter named LSFC and LNN. Additionally, only selective inputs variables which are assumed to have significant variability on SFC and LNN are considered.

The full quadratic model is given by

17 $$\begin{aligned} \check{R} =r_{0}+r_{1}A+r_{2}B+r_{3}C+r_{11}A^{2}+r_{22}B^{2}+r_{33}C^{2}+r_{12}AB+r_{13}AC+r_{23}BC, \end{aligned}$$

involving intercept, quadratic, linear and two-factor bilinear terms. Thus $\check{R}$ defines local response of SFC and NN. It consists of three independent input parameters coded via $\left( A,B,C\right)$ symbols (solid volume fraction, inertia coefficient and permeability parameter respectively) for skin friction and $\left( A_{1},B_{1},C_{1}\right)$ (solid volume fraction, radiation and Biot parameters) for LNN (For simplicity for LNN we also use same symbols *A*,  *B*,  and *C*). According to RSM, twenty runs along with 19, DOF are suitable for chosen 3 stages of parameters. These quantities are small, medium and large as $(-1,0,1)$.

Table 4 shows input parameters according to its respective levels and symbols. In addition, CCD (Central Composite Design) for conduct of a numerical experiment is commonly used in $R-$programming. The series of twenty runs of experiments is planned to refer the term of $2^{F}+2F+P,$ where $P=6$ is center points number and $F=3$ is number of factors. The sequence of experimental programs is given for SWCNT-Water, MWCNT-Water, SWCNT-Ethylene glycol, MWCNT-Ethylene glycol in Tables 4 and 5 for both SFC and LNN respectively. ANOVA is a statistical strategic significance for utility of uncertainty in dependency of defined variables on RSM model. ANOVA studies the RSM model’s optimization criterion for degree of model accuracy by which numerical estimators are DOF, SS, MMS, $F-$value and $p-$ value. Tables 6, 7, 8 and 9 demonstrate ANOVA analysis to point out corelations among SFC and LNN numbers to three independent input parameters for SWCNT and MWCNT for both type of base fluids.

Sensitivity is extensively described in terms of model variables as derivative of response function. Sensitivity research explores the eccentric prerequisites provided by model output assigned by input variables, that compared to estimation of model vigor.

Consequently, mathematical Eq. (17) related to SFC and LNN may be rewritten according to SWCNT-Water, MWCNT-Water, SWCNT-Ethylene glycol and MWCNT-Ethylene glycol respectively as

18 $$\begin{aligned} C_{fr}^{1}= & \,0.1132673-0.2071897A+0.188478B+0.387765C+3.192259A^{2}+0.004457B^{2} \\&+0.057906C^{2}-0.259289AB-0.576470AC+0.008518BC, \end{aligned}$$

19 $$\begin{aligned} C_{fr}^{2}= & \, 0.085376-2.768411A+0.146517B+0.403900C+2.832044A^{2}+0.004853B^{2} \\&+0.104061C^{2}-0.265042AB-0.531551AC-0.069695BC, \end{aligned}$$

20 $$\begin{aligned} C_{fr}^{3}= & \, 10.544045+10.543954A+0.587996B+6.692676C+7.053726A^{2}-0.004784B^{2} \\&-0.018624C^{2}+0.125535AB+6.629870AC+0.003585BC, \end{aligned}$$

21 $$\begin{aligned} C_{fr}^{4}= & \, 8.918313+9.656962A+0.450119B+6.645453C+7.302046A^{2}-0.001389B^{2} \\&-0.016389C^{2}+0.027179AB+6.585451AC+0.003314BC, \end{aligned}$$

22 $$\begin{aligned} Nu_{r}^{1}= & \, 1.32610+3.54561A+0.03656B+1.68461C+2.92760A^{2}-0.02414B^{2} \\&-0.04400C^{2}-0.02013AB+1.64936AC+0.01518BC, \end{aligned}$$

23 $$\begin{aligned} Nu_{r}^{2}= & \, 1.34639+4.91565A+0.04513B+2.14538C+4.29979A^{2}-0.04480B^{2} \\&-0.06517C^{2}-0.01223AB+2.22975AC+0.01770BC, \end{aligned}$$

24 $$\begin{aligned} Nu_{r}^{3}= & \, 2.456253+5.450058A+0.120914B+2.860162C+4.108897A^{2}-0.001763B^{2} \\&-0.004053C^{2}-0.006705AB+2.716343AC+0.064780BC, \end{aligned}$$

25 $$\begin{aligned} Nu_{r}^{4}= & \, 2.375163+5.540966A+0.128634B+2.879130C+4.256968A^{2}-0.001772B^{2} \\&-0.003932C^{2}-0.001193AB+2.762033AC+0.069080BC. \end{aligned}$$

### Sensitivity analysis

The partial derivative of response function according to model’s parameters is named as sensitivity. Consequently, sensitivity function of SFC and for LNN are defined in relation to governing variables, (Casson fluid parameter $\left( A\right)$, inertia coefficient $\left( B\right)$, permeability parameter $\left( C\right)$ corresponds to SFC and solid volume fraction $\left( A\right)$, radiation parameter $\left( B\right)$ and Biot parameter $\left( C\right)$ corresponds to LNN) relying on (18–25).

For SWCNT-Water correspond to SFC

26 $$\begin{aligned} \frac{\partial C_{fr}^{1}}{\partial A}= -0.2071897+6.384518A-0.259289B-0.576470C, \end{aligned}$$

27 $$\begin{aligned} \frac{\partial C_{fr}^{1}}{\partial B}= 0.188478+0.008914B-0.259289A+0.008518C, \end{aligned}$$

28 $$\begin{aligned} \frac{\partial C_{fr}^{1}}{\partial C}= 0.387765+0.115812C-0.576470A+0.008518B. \end{aligned}$$

For MWCNT-Water correspond to SFC

29 $$\begin{aligned} \frac{\partial C_{fr}^{2}}{\partial A}= -2.768411+5.664088A-0.265042B-0.531551C, \end{aligned}$$

30 $$\begin{aligned} \frac{\partial C_{fr}^{2}}{\partial B}= 0.146517+0.009706B-0.265042A-0.069695C, \end{aligned}$$

31 $$\begin{aligned} \frac{\partial C_{fr}^{2}}{\partial C}= 0.403900+0.208122C-0.531551A-0.069695B. \end{aligned}$$

For SWCNT-Ethylene glycol correspond to SFC

32 $$\begin{aligned} \frac{\partial C_{fr}^{3}}{\partial A}= 10.543954+14.107452A+0.125535B+6.629870C, \end{aligned}$$

33 $$\begin{aligned} \frac{\partial C_{fr}^{3}}{\partial B}= 0.587996-0.009568B+0.125535A+0.003585C, \end{aligned}$$

34 $$\begin{aligned} \frac{\partial C_{fr}^{3}}{\partial C}= 6.692676-0.037248C+6.629870A+0.003585B. \end{aligned}$$

For MWCNT-Ethylene glycol correspond to SFC

35 $$\begin{aligned} \frac{\partial C_{fr}^{4}}{\partial A}= 9.656962+14.604092A+0.027179B+6.585451C, \end{aligned}$$

36 $$\begin{aligned} \frac{\partial C_{fr}^{4}}{\partial B}= 0.450119-0.002778B+0.027179A+0.003314C, \end{aligned}$$

37 $$\begin{aligned} \frac{\partial C_{fr}^{4}}{\partial C}= 6.645453-0.032778C+6.585451A+0.003314B. \end{aligned}$$

For SWCNT-Water correspond to LNN

38 $$\begin{aligned} \frac{\partial Nu_{r}^{1}}{\partial A}= 3.54561+5.8552A-0.02013B+1.64936C, \end{aligned}$$

39 $$\begin{aligned} \frac{\partial Nu_{r}^{1}}{\partial B}= 0.03656-0.04828B-0.02013A+0.01518C, \end{aligned}$$

40 $$\begin{aligned} \frac{\partial Nu_{r}^{1}}{\partial C}= 1.68461-0.0880C+1.64936A+0.01518B. \end{aligned}$$

For MWCNT-Water correspond to LNN

41 $$\begin{aligned} \frac{\partial Nu_{r}^{2}}{\partial A}= 4.91565+8.59958A-0.01223B+2.22975C, \end{aligned}$$

42 $$\begin{aligned} \frac{\partial Nu_{r}^{2}}{\partial B}= 0.04513-0.0896B-0.01223A+0.01770C, \end{aligned}$$

43 $$\begin{aligned} \frac{\partial Nu_{r}^{2}}{\partial C}= 2.14538-0.13034C+2.22975A+0.01770B. \end{aligned}$$

For SWCNT-Ethylene glycol correspond to LNN

44 $$\begin{aligned} \frac{\partial Nu_{r}^{3}}{\partial A}= 5.450058+8.217794A-0.006705B+2.716343C, \end{aligned}$$

45 $$\begin{aligned} \frac{\partial Nu_{r}^{3}}{\partial B}= 0.120914-0.003526B-0.006705A+0.064780C, \end{aligned}$$

46 $$\begin{aligned} \frac{\partial Nu_{r}^{3}}{\partial C}= 2.860162-0.008106C+2.716343A+0.064780B. \end{aligned}$$

For MWCNT-Ethylene glycol correspond to LNN

47 $$\begin{aligned} \frac{\partial Nu_{r}^{4}}{\partial A}= 5.540966+8.513936A-0.001193B+2.762033C, \end{aligned}$$

48 $$\begin{aligned} \frac{\partial Nu_{r}^{4}}{\partial B}= 0.128634-0.003544B-0.001193A+0.069080C, \end{aligned}$$

49 $$\begin{aligned} \frac{\partial Nu_{r}^{4}}{\partial C}= 2.879130-0.007864C+2.762033A+0.069080B. \end{aligned}$$

## Discussion

The governing transformed differential system (9–11) with boundary conditions (12) are solved via Runge–Kutta Fehlberg technique. The boundary layer thickness $\eta _{\infty }$ is putting 10. Tables  2 and 3 show numerical values of SFC and LNN correspond to SWCNT and MWCNT by considering water and Ethylene glycol as base fluid, for various values of $\phi ,$ $\gamma _{1},$ $k_{1},$ $F_{r},$ $M_{1},$ $\lambda _{1},$ $R_{*},$ *Ec* and $\gamma _{2}$.

Tables 7, 8, 9 and 10 are related to ANOVA study, to set up correlations among SFC and LNN to independent input factors. In study of ANOVA, *F*-value is estimation of data variance over average value, whereas *p*-value is probability validation of model accuracy from statistical context. High *F*-value labels a significant outcome while small *p*-value shows sufficient support to significance of outcome. Therefore, *F*-value is often utilized to offer sufficient evidence on the importance of outcome alongside the $p-$ value. Accordingly, effect of linear, two-factor bilinear and square terms are known to be statistically meaningful for response parameters (SFC and LNN), with good evidence of high $F-$value and low *p*-value.

**Figure 2:**
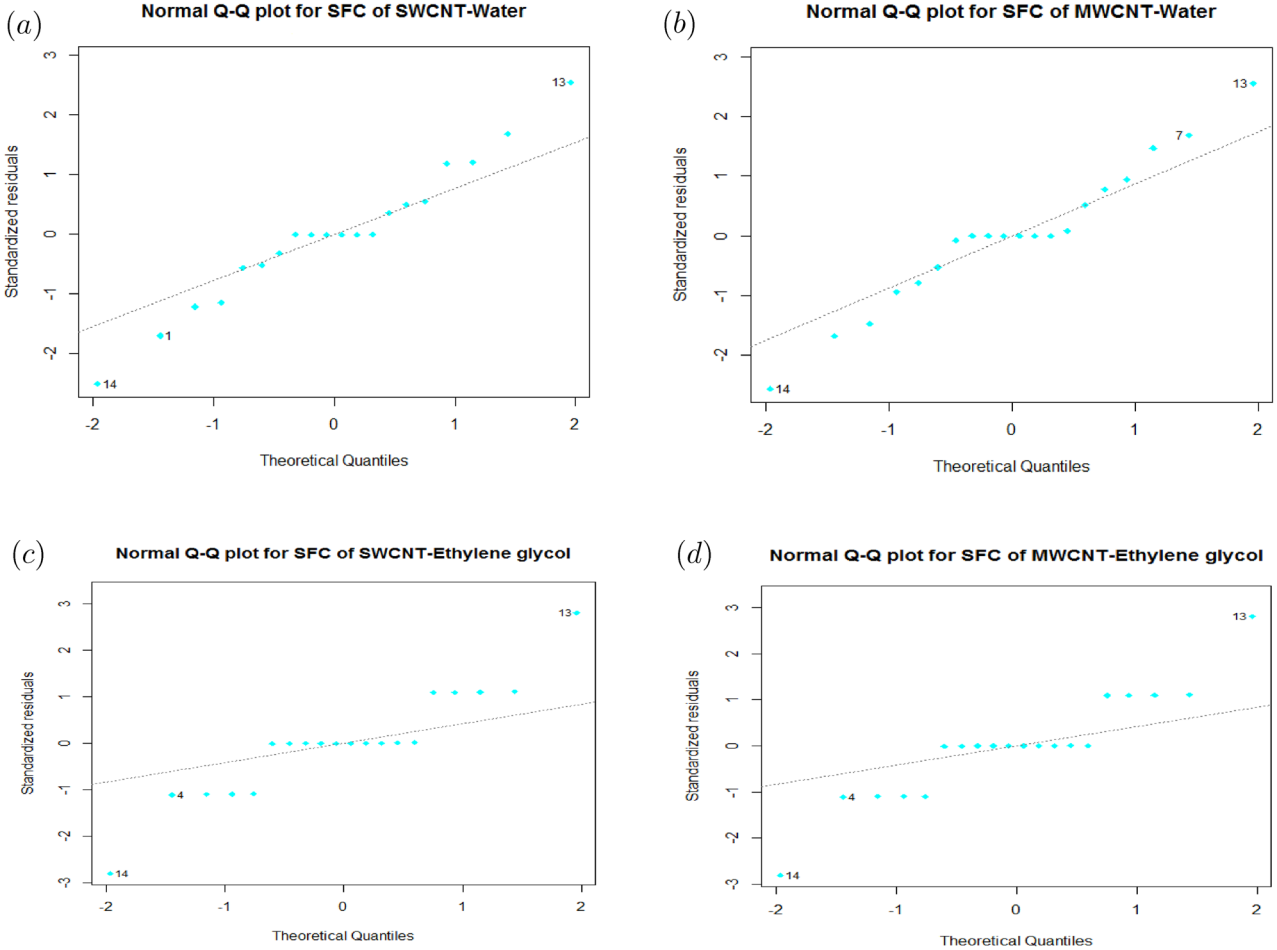
(**a**) Residuals Q–Q graph of SFC for SWCNT-Water. (**b**) Residuals Q–Q graph of SFC for MWCNT-Water. (**c**) Residuals Q–Q graph of SFC for SWCNT-Ethylene glycol. (**d**) Residuals Q–Q graph of SFC for MWCNT-Ethylene glycol.

**Figure 3:**
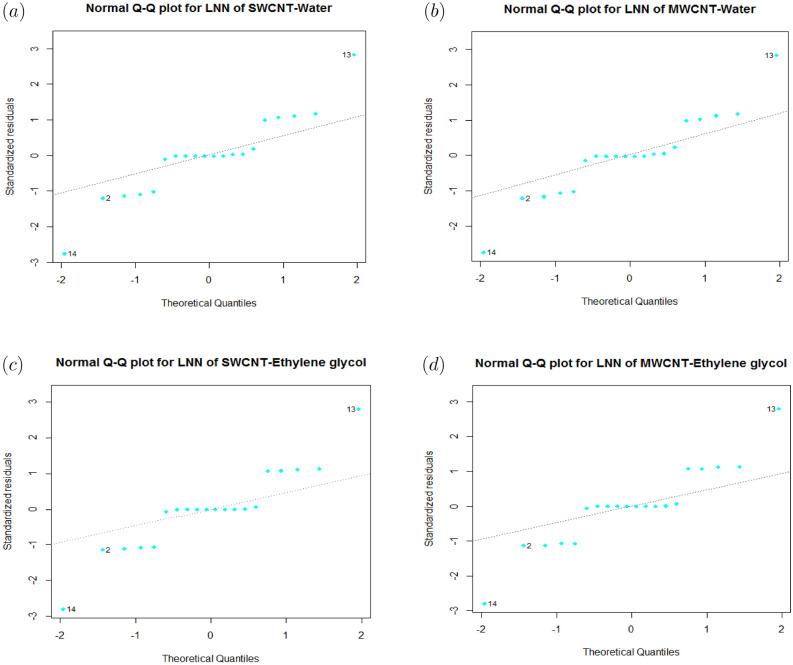
(**a**) Residuals Q–Q graph of LNN for SWCNT-Water. (**b**) Residuals Q-Q graph of LNN for MWCNT-Water. (**c**) Residuals Q–Q graph of LNN for SWCNT-Ethylene glycol. (**d**) Residuals Q–Q graph of LNN for MWCNT-Ethylene glycol.

Particularly, residual error is unspecified data point via regression line, whereas lack of fit depicts if model neglects to display functional connectedness between input and output response. Figs. 2 and 3 show normal $Q{-}Q$ residual plot for SFC and LNN correspond to SWCNT and MWCNT (Base fluid: Water and Ethylene glycol). The plots that appear with a straight line indicating the errors are normally distributed. Hence, regression model is properly fitted.

Regression coefficients for responses (SFC and LNN) via its corresponding p-value for non-linear polynomial model in (17) are given in Tables 11, 12, 13 and 14 for SFC and LNN corresponds to SWCNT and MWCNT (Base fluid: Water and Ethylene glycol). It is noteworthy that large $p-$value is considered to be statistically insignificant, indicating no relative change in output can be noted due to change in input. Further, a term with low $p-$value $(\le 0.05)$ that is statistically important elsewhere can be overlooked. As a consequence, *A*, $A^{2}$, *AB*,  *AC* *B*, and *C* corresponds to SWCNT-Water while *A*,  *C*,  $A^{2},$ *AB* and *AC* corresponds to MWCNT-Water are significant factors for SFC. On the other hand, *A*, *C*, $A^{2}$ and *AC* corresponds to SWCNT-Ethylene glycol while *A*,  *C*,  $A^{2}$ and *AC* corresponds to MWCNT-Ethylene glycol are significant factors for SFC. For LNN, *A*, *C*, $A^{2}$ and *AC* corresponds to both SWCNT and MWCNT (Water as base fluid) are important terms. On the other side, same terms are important for both SWCNT and MWCNT when Ethylene glycol is considered as base fluid.

Additionaly, the values of $R^{2}$ and $R^{2}-adj={\bar{R}}^{2}$, are also dispensed in Tables 11, 12, 13 and 14. It offers comprehensive details on the RSM model’s $``goodness-of-fit''$. This is noticed that SFC and LNN are identified with higher $R^{2}$ and ${\bar{R}}^{2}=R^{2}-adj$ values $(99.68\%,$ $99.4\%,$ $99.46\%,$ $98.98\%,$ $98.22\%,$ $96.62\%,$ $98.08\%,$ $96.36\%,$ $98.7\%,$ $97.53\%,$ $98.62\%,$ $97.37\%,$ $98.73\%,$ $97.59\%,$ $98.69\%$ and $97.51\%$ correspond to SWCNT and MWCNT (Base fluid: Water and Ethylene glycol), respectively) present a accurately predicted correlation among regressand and regressor.

**Figure 4:**
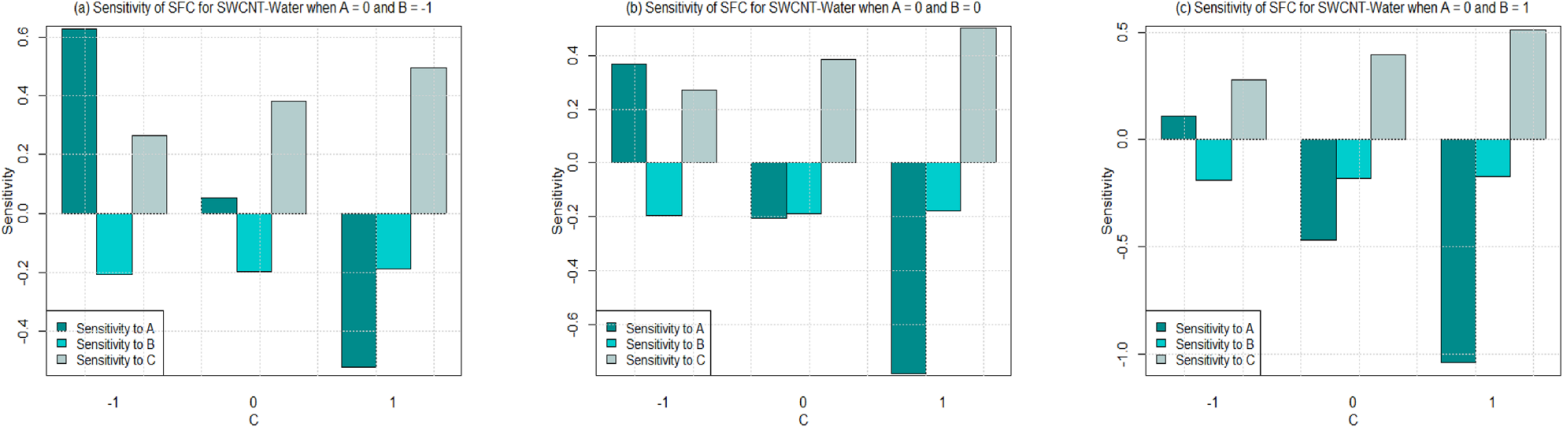
Sensitivity results for the SFC for SWCNT-Water.

**Figure 5:**
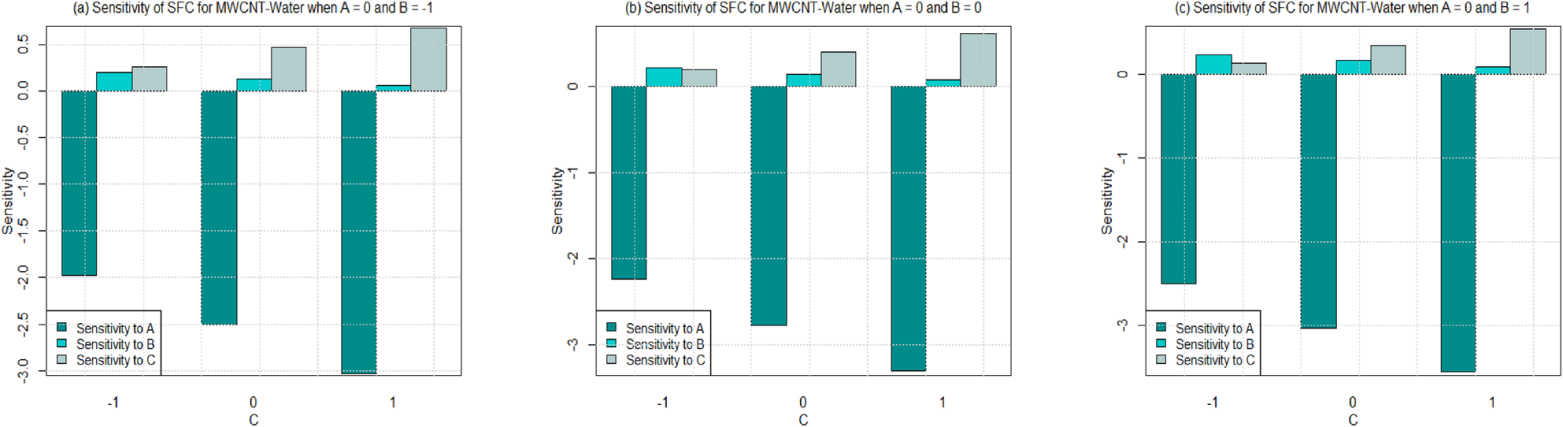
Sensitivity results for the SFC for MWCNT-Water.

**Figure 6:**
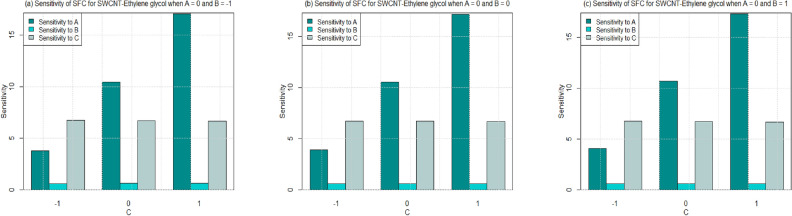
Sensitivity results for the SFC for SWCNT-Ethylene glycol.

**Figure 7:**
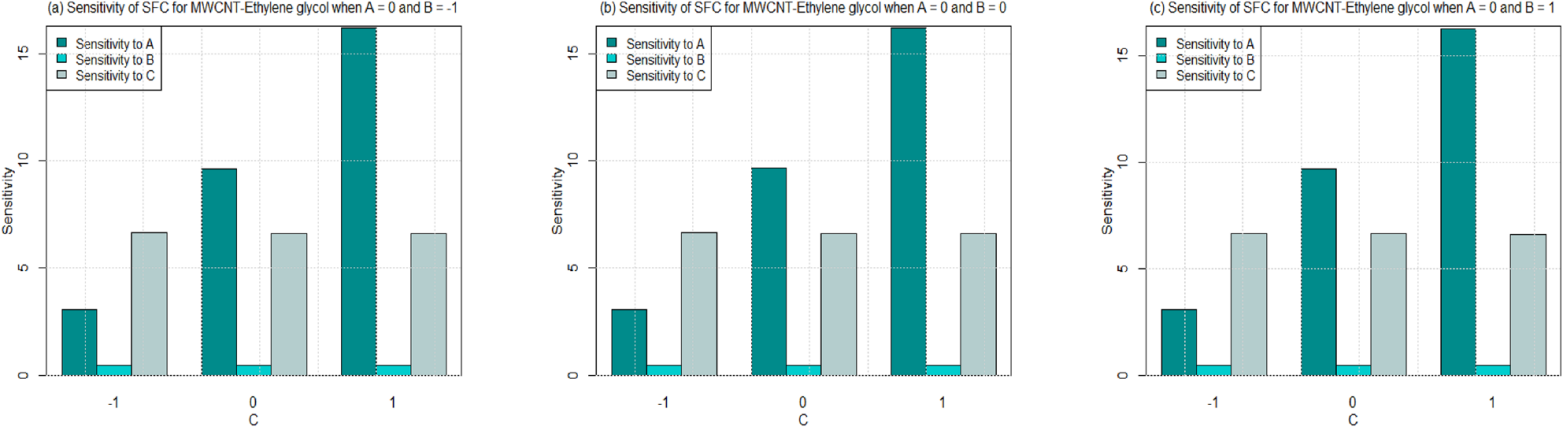
Sensitivity results for the SFC for MWCNT-Ethylene glycol.

**Figure 8:**
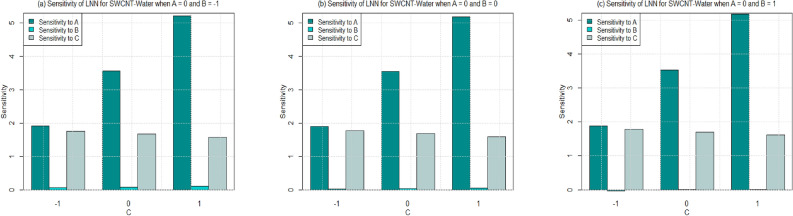
Sensitivity results for the LNN for SWCNT-Water.

**Figure 9:**
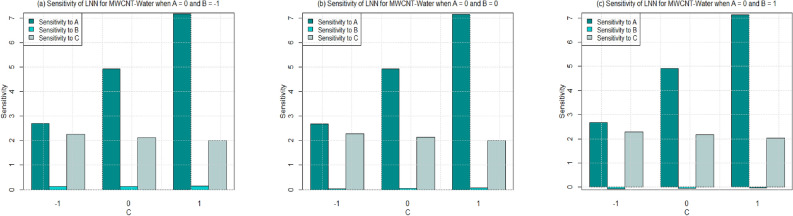
Sensitivity results for the LNN for MWCNT-Water.

**Figure 10:**
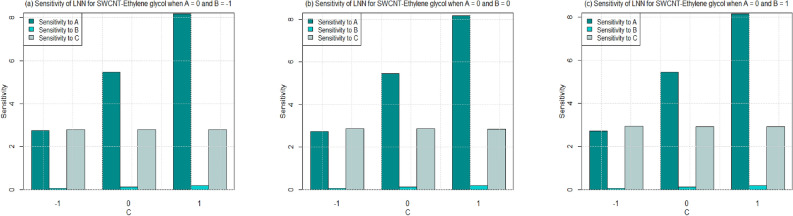
Sensitivity results for the LNN for SWCNT-Ethylene glycol.

**Figure 11:**
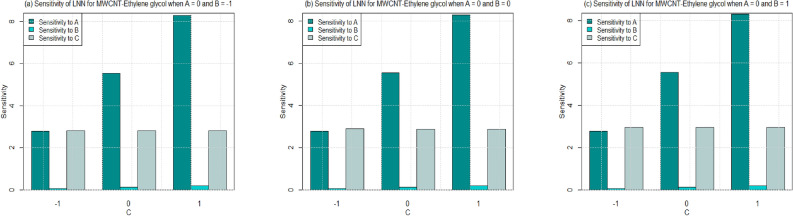
Sensitivity results for the LNN for MWCNT-Ethylene glycol.

Utilizing $\left( 26-49\right)$, the sensitivity outcomes of SFC and LNN corresponding to regressors (*A*, *B*, and *C*) are ascertained (see Tables 15 and 16) correspond to SWCNT and MWCNT when water and Ethylene glycol are considered as base fluid. It is noticed that a $+ve$ sensitivity esteem showing increase of regressor induces an increment within the response function and conversely for $-ve$ sensitivity. For a more noteworthy insight, the sensitivity results were sketched into Bar Charts (Figs. 4, 5, 6, 7, 8, 9, 10 and 11).

From this viewpoint, an upright bar showing a $+ve$ sensitivity and an inverted bar showed $-ve$ sensitivity. Figure 4 shows the sensitivity outcomes for SFC via SWCNT for the case where water is taken as base fluid. The overall trend shows that the sensitivity of the SFC rises with increment in governing variables under all values of permeability parameter. However, the sensitivity of the SFC via SWCNT becomes higher by increasing values of permeability number from 0.2 to 0.6 i.e. $C=-1$ to 1. Additionally, sensitivity of SFC via SWCNT-water towards the permeability number is higher than the solid volume fraction for $C=0$ and $C=1$. For the case of lower permeability number $\left( C=-1\right)$, the SFC (SWCNT-water) seems to have a high sensitivity towards the solid volume fraction instead of permeability and inertia coefficient (see Fig. 4a,b). On the other side, it is noted from Fig. 4c that the SFC (SWCNT-water) has a higher sensitivity for permeability as compare to solid volume fraction and inertia coefficient for $C=-1$.

Sensitivity of SFC via MWCNT for the case where water is taken as base fluid at various values of permeability parameter is shown in Figs. 5a–c. It is noted that the sensitivity of SFC via MWCNT for the case where water is taken as base fluid falls with the increment in parameters under all values of solid volume fraction. Although, the SFC (MWCNT-water) have a low sensitivity corresponds to inertia coefficient for the increasing values of permeability as well as inertia coefficient. Additionally, it is observed that the sensitivity of SFC via MWCNT-water have a higher sensitivity towards permeability parameter as compare to inertia and solid volume fraction for $C=0$ and $C=1$ (see Fig. 5a–c). Furthermore, for the lower permeability parameter i.e. $C=-1$, the sensitivity of SFC towards inertia is higher instead of permeability and solid volume fraction (see Fig. 5b,c). Whereas, sensitivity of SFC is lower towards inertia instead of permeability and higher in case of solid volume fraction (see Fig. 5a).

Figures  6 and 7 are ploted to see the sensitivity of SFC for both the cases SWCNT and MWCNT when Ethylene-glycol is used as a base fluid. In Fig. 6, the sensitivity of SFC (SWCNT-Ethylene-glycol) towards volume fraction is higher for increasing permeability as well as inertia coefficient. It is also noted that the sensitivity of SFC (SWCNT-Ethylene-glycol) is approximately equal for permeability under all levels of permeability number (see Fig. 6a–c). Meanwhile, same pattern is noted for inertia under all levels of permeability. Similar behaviour is observed for sensitivity of SFC (MWCNT) when Ethylene-glycol is taken as base fluid (Fig. 7).

Sensitivity of LNN via SWCNT and MWCNT by considering two type of base fluids towards various parameter at different levels of Biot number are plotted in Figs. 8, 9 and 11. In general, the sensitivity of LNN via SWCNT-water towards solid volume fraction increases by increasing Biot number. But the sensitivity of LNN (SWCNT) towards Biot is approximately equal despite the increment in Biot and radiation parameter. Figure 8a shows the similar positive sensitivity at low radiation parameter under all levels of Biot. However, very small sensitivity of LNN towards radiation is noted at higher Biot number ($C=1$) see Fig. 8b–c.

Figure 9 is drawn to see the sensitivity results for the LNN for MWCNT and water is taken as base fluid. Overall, trend noted from figures 9a–c shows that sensitivity of the LNN that rises with increment in parameters under all values of Biot number. Yet, sensitivity of LNN remains approximately constant with increasing Biot number from 0.2 to 0.6 ($C=-1$ to 1). Additionally, the sensitivity of LNN towards the Solid volume fraction is higher than the radiation and Biot number for $C=-1,0,1$ (see Figs. 9a–c. Similar behavior is noted for Figs. 10 and 11 for both types of carbon nanotubes, when base fluid is Ethylene glycol. Only for the case of lowest Biot number the LNN seems to have a higher sensitivity towards Biot number instead of radiation and solid volume fraction.

**Figure 12:**
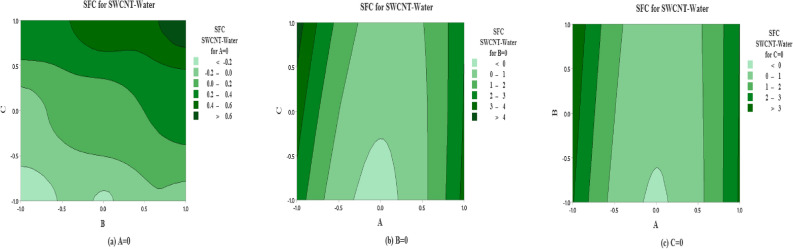
Predicted responses as a function of factors for SFC for SWCNT-Water, expressed coded level, showing the effects of (**a**)  *B* and *C*  (**b**) *A* and *C* and (**c**)  *A* and *B*.

The predicted non-dimensional SFC as a function of the solid volume fraction $\left( A\right)$, inertia coefficient $\left( B\right)$ and permeability parameter $\left( C\right)$ are shown in Fig. 12 for SWCNT-Water. The effects of inertia coefficient and permeability parameter on non-dimensional SFC for $A=0$ $\left( \phi =0.1\right)$ are shown in Fig.  12a. It is noted that maximum non-dimensional SFC occurs near higher level for inertia coefficient $\left( B\right)$ and permeability parameter $\left( C\right)$ and vice versa. On the other side, the maximum average of SFC occurs near the high and low levels for solid volume fraction $\left( A\right)$ and inertia coefficient $\left( B\right)$ (see Fig. 12b). But the moderate value of SFC occurs at the middle levels for solid volume fraction $\left( A\right)$. Moreover same behavior is observed in Fig. 12c.

**Figure 13:**
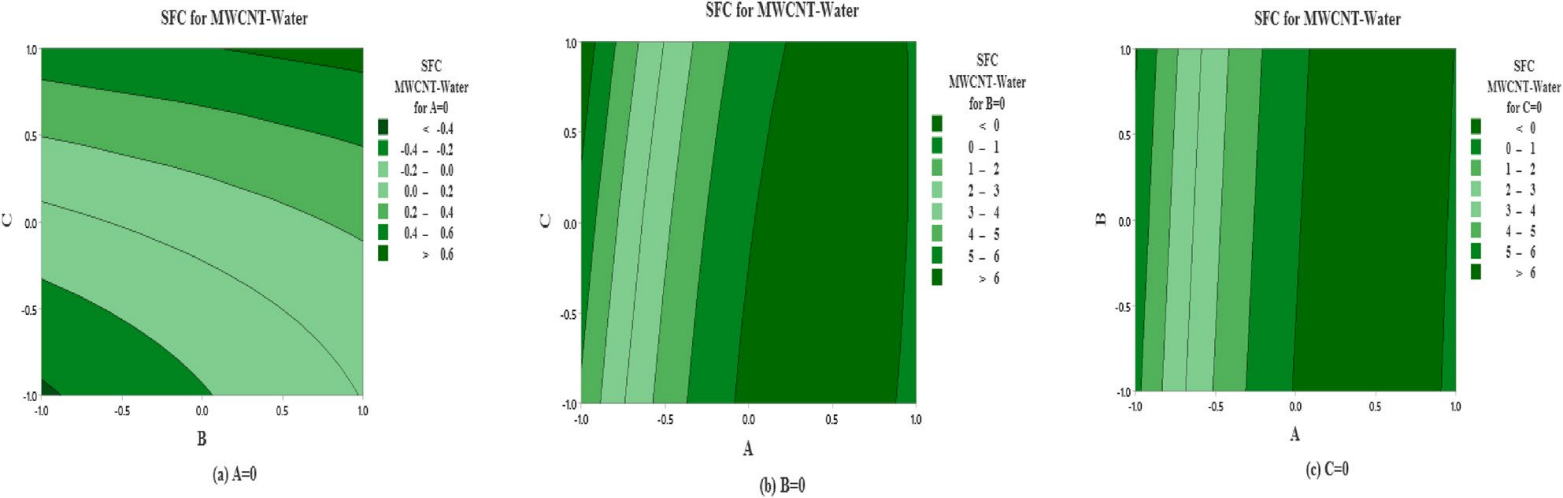
Predicted responses as a function of factors for SFC for MWCNT-Water, expressed coded level, showing the effects of (**a**)  *B* and *C*  (**b**) *A* and *C* and (**c**) *A* and *B*.

The predicted SFC as a function of solid volume fraction $\left( A\right)$, inertia coefficient $\left( B\right)$ and permeability number $\left( C\right)$ are plotted in Fig. 13 for MWCNT-Water. The effects of inertia coefficient and permeability number on non dimensional SFC for $A=0$ $\left( \phi =0.1\right)$ are shown in Fig. 13a. It is noted that maximum non-dimensional SFC occurs near large level for inertia coefficient $\left( B\right)$ and higher and lower levels for permeability parameter $\left( C\right)$. On the other side, the moderate level of SFC occurs near the extreme level of inertia coefficient (*B*) and the moderate level of permeability coefficient $\left( C\right) .$ In addition, the maximum average of SFC is observed at the extreme levels of *A* and *C*, on the contrary the oposite behaviour observed for lower levels of *A* and *C* and low levels for solid volume fraction $\left( A\right)$ and inertia coefficient $\left( B\right)$ (see Fig. 13b). Also the same pattern is observed in Fig. 13c.

**Figure 14:**
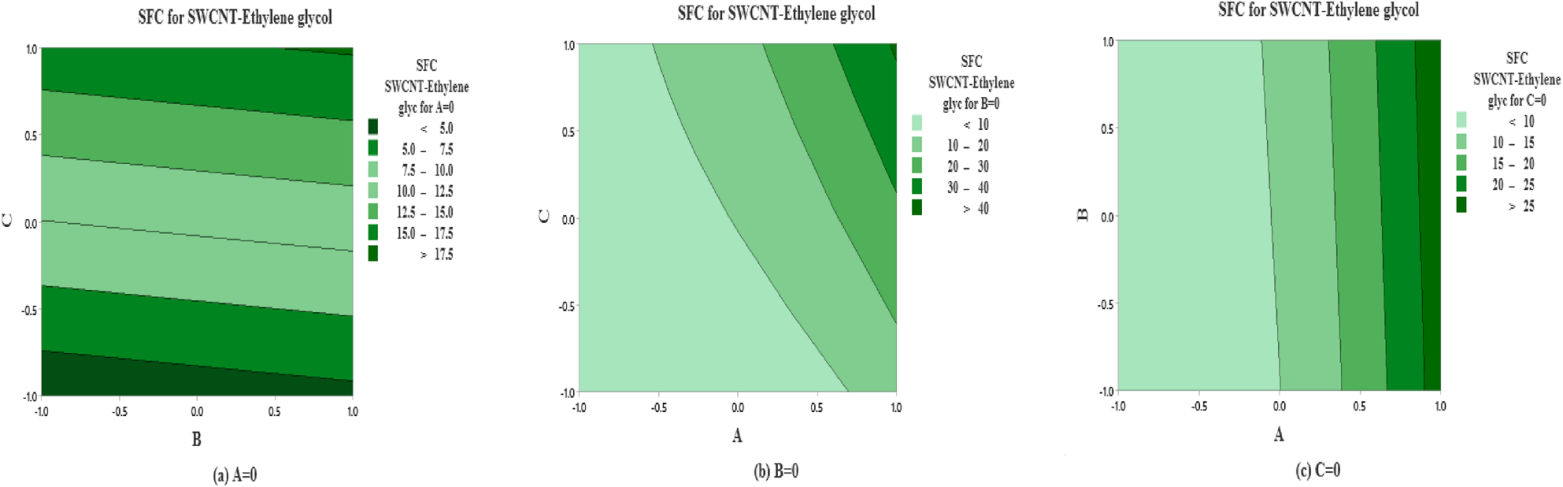
Predicted responses as a function of factors for SFC for SWCNT-Ethylene glycol, expressed coded level, showing the effects of (**a**)  *B* and *C*  (**b**) *A* and *C* and (**c**)  *A* and *B*.

The predicted non-dimensional SFC as a function of *A*, *B* and *C* are shown in Fig. 14 for the case of SWCNT and Ethylene glycol is taken as base fluid. The impact of inertia coefficient and permeability parameter on non-dimensional SFC for solid volume fraction $(A=0$ $\left( \phi =0.1\right) )$ are shown in Fig. 14a for SWCNT-Ethylene glycol. It is noted that maximum non-dimensional SFC is noted near all the levels for inertia coefficient $\left( B\right)$ and extreme high and low levels for permeability parameter $\left( C\right)$. On the other hand, moderate behavior is observed at the moderate level of C and all the levels of A. In Fig. 14b, at the extremely higher level of *A* and *C* the maximum non-dimensional SFC is reflected. In addition the maximum average SFC is examined near the extreme level for solid volume fraction $\left( A\right)$ and all levels for inertia coefficent $\left( B\right)$ (see 14(*c*)).

**Figure 15:**
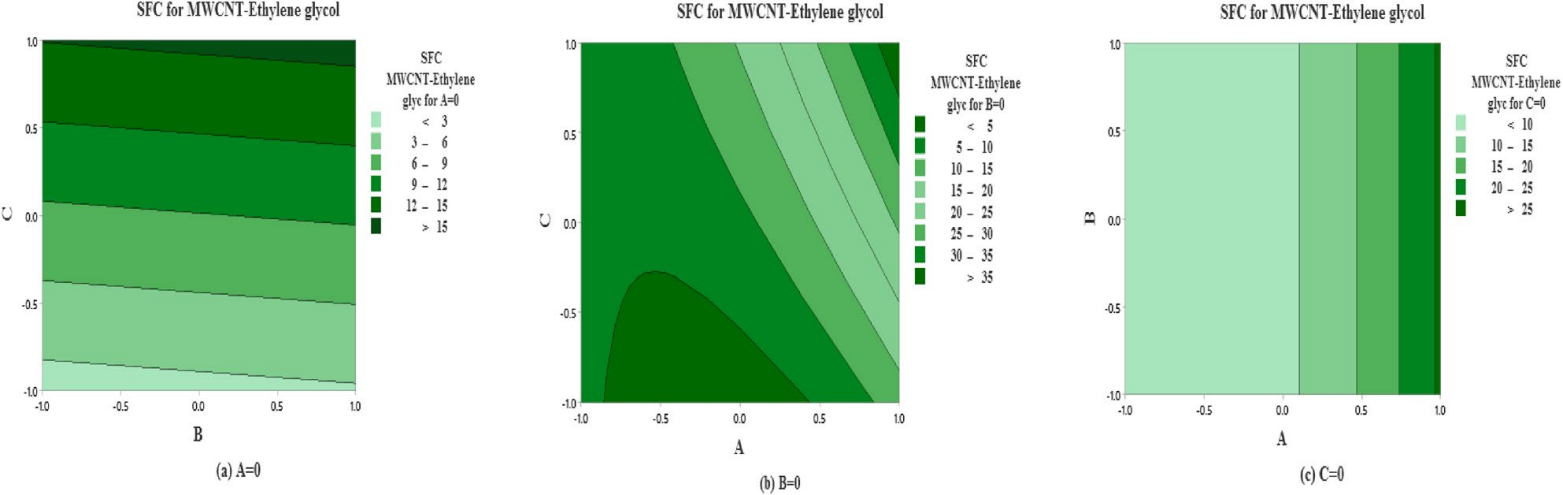
Predicted responses as a function of factors for SFC for MWCNT-Ethylene glycol, expressed coded level, showing the effects of (**a**)  *B* and *C*  (**b**)  *A* and *C* and (**c**) *A* and *B*.

The predicted non-dimensional SFC density as a function of solid volume fraction $\left( A\right)$, inertia coefficient $\left( B\right)$ and permeability parameter $\left( C\right)$ are analyzed in Fig. 15 for the case of MWCNT and Ethylene glycol is taken as base fluid. The strenght of inertia coefficient and permeability parameter on non-dimensional SFC (MWCNT-Ethylene glycol) for $A=0$ are drawn in Fig. 15(a). It is indicated that average maximum non-dimensional SFC (MWCNT-Ethylene glycol) is examined at the extreme level of *C* and all levels of *B*. While the maximum level of non-dimensional SFC for MWCNT-Ethylene glycol is noted on moderate level of *A* and higher level of permeability parameter $\left( C\right)$ (see 15(*b*)). In addition the maximum average SFC (MWCNT-Ethylene glycol) is analyzed near the higher level for solid volume fraction $\left( A\right)$ and all levels for inertia coefficent $\left( B\right)$ (see 15(*c*)).

**Figure 16:**
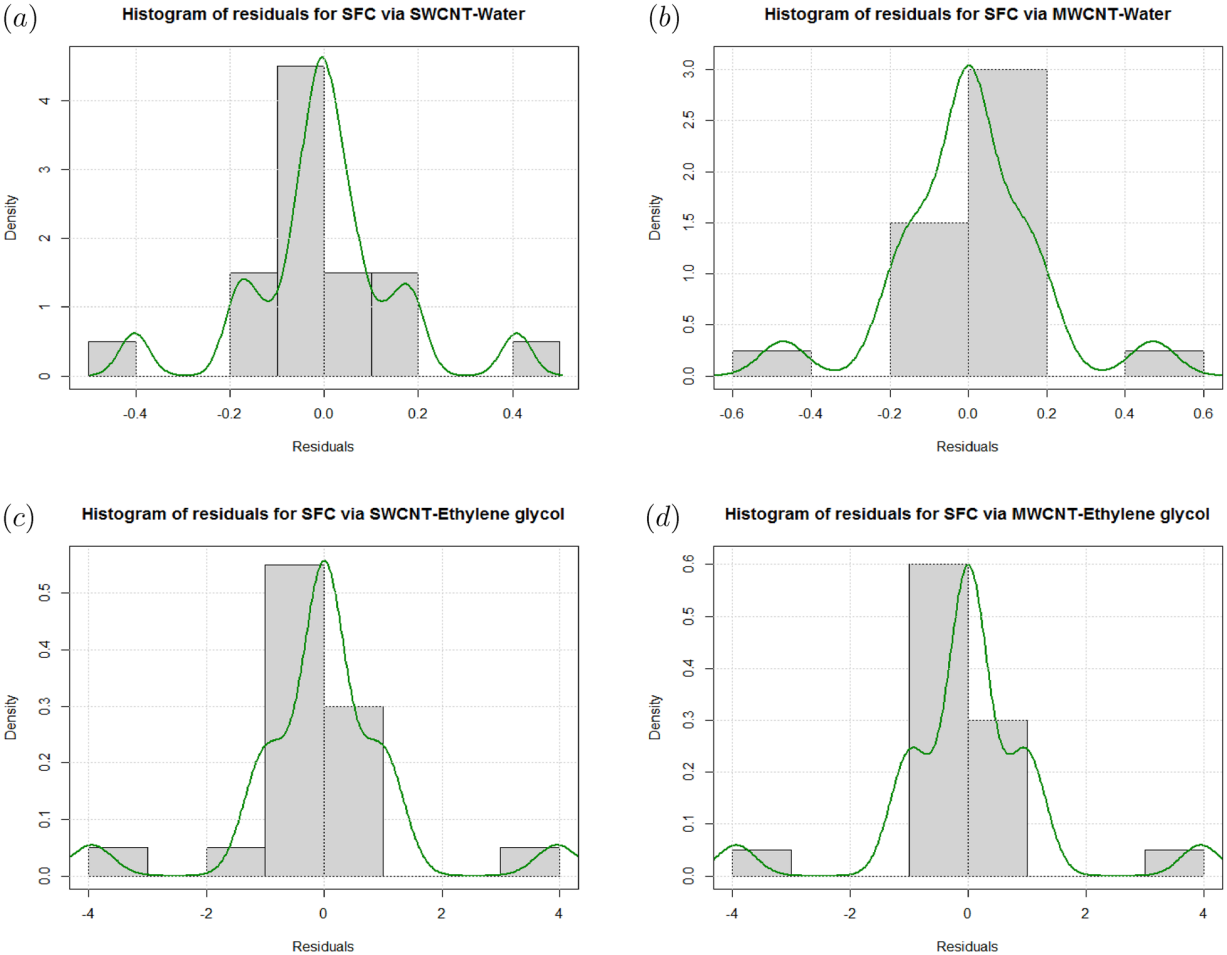
(**a**) Histogram and density plots for residuals of CFC via SWCNT-Water. (**b**) Histogram and density plots for residuals of CFC via MWCNT-Water. (**c**) Histogram and density plots for residuals of CFC via SWCNT-Ethylene glycol. (**d**) Histogram and density plots for residuals of CFC via MWCNT-Ethylene glycol.

**Figure 17:**
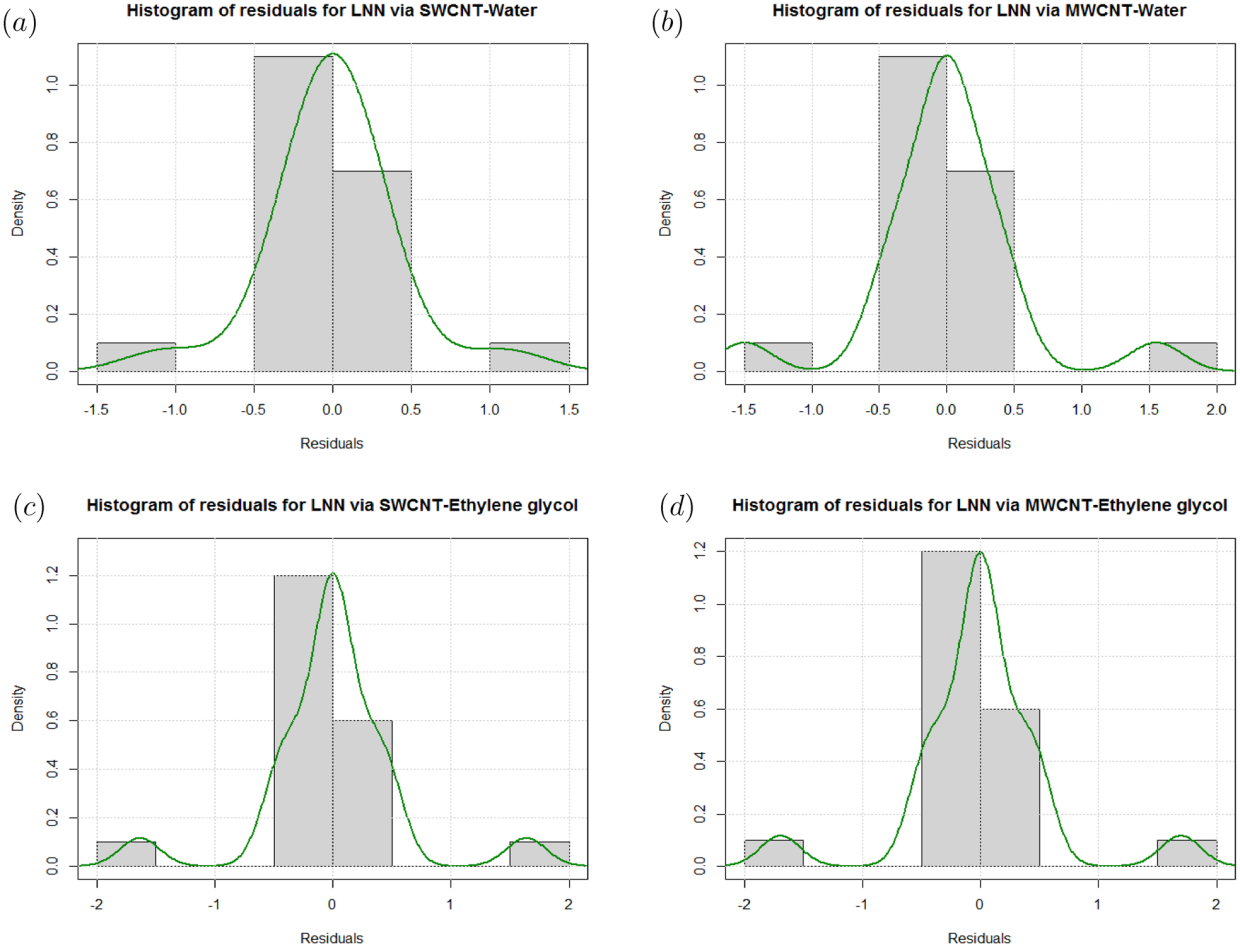
(**a**) Histogram and density plots for residuals of LNN via SWCNT-Water. (**b**) Histogram and density plots for residuals of LNN via MWCNT-Water. (**c**) Histogram and density plots for residuals of LNN via SWCNT-Ethylene glycol. (**d**) Histogram and density plots for residuals of LNN via MWCNT-Ethylene glycol.

In Figs. 16 and 17, residual histograms along with the density function are shown for both local SFC and NN via SWCNT and MWCNT using water and Ethylene glycol as a base fluids. It is noted from these figures that behavior of the residual histogram is less skewed distribution and shown the behaviors which are almost similar to a symmetrical distribution. The results in Table 17 were determined to validate the current results with previously reported results. In this case, we can see that the current numerical solution agrees with previous solution by^46^ in a limited context.

**Table 2:** The values of SFC and LNN for various values of physical parameters when $S_{1}=0.6,$ $\delta _{1}=0.7.$

$\phi$	$\gamma _{1}$	$k_{1}$	$F_{r}$	$M_{1}$	$\lambda _{1}$	$R_{*}$	*Ec*	$\gamma _{2}$	$\left( \mathrm {Re}_{r}\right) ^{1/2}C_{fr}$			
									S-W	M-W	S-EG	M-EG
0.0	0.5	0.2	2.5	0.5	0.2	2	0.3	0.3	5.8657	5.8657	6.04769	6.04769
0.3									7.3139	5.8586	8.15695	6.73657
0.6									8.1836	5.5902	12.5023	10.1601
0.3	0.1	0.2	2.5	0.5	0.2	2	0.3	0.3	10.555	9.33604	11.7627	10.5817
	0.3								7.6994	6.33141	8.58427	7.25390
	0.6								7.2520	5.76426	8.08962	6.63538
0.3	0.5	0.0	0.2	0.5	0.2	2	0.3	0.3	5.47992	4.04873	6.19621	4.79524
		0.2							7.31395	5.85859	8.15695	6.73657
		0.4							9.14079	7.65876	10.0999	8.65695
0.3	0.5	0.2	2.0	0.5	0.2	2	0.3	0.3	6.7388	5.40471	7.59540	6.28623
			2.5						7.31395	5.85859	8.15695	6.73657
			3.0						7.89026	6.31333	8.71908	7.18734
0.3	0.5	0.2	2.5	0.0	0.2	2	0.3	0.3	7.00101	5.54991	7.82330	6.40650
				0.5					7.31395	5.85859	8.15695	6.73657
				1.0					8.25125	6.78253	9.15471	7.72306
0.3	0.5	0.2	2.5	0.5	$\lambda _{1}0.0$	2	0.3	0.3	9.62859	7.94729	9.15900	7.66965
					0.3				6.36505	4.98895	7.67656	6.28952
					0.6				3.98682	2.79522	6.31458	5.02364
0.3	0.5	0.2	2.5	0.5	0.3	$R_{*}0$	0.3	0.3	6.39614	5.02085	7.70083	6.31029
						1			6.37768	5.00214	7.68839	6.29965
						2			6.36505	4.98895	7.67656	6.28952
0.3	0.5	0.2	2.5	0.5	0.3	2	0.0	0.3	9.56666	7.89144	9.15877	7.66945
							0.3		6.36505	4.98895	7.67656	6.28952
							0.6		4.01771	2.82226	6.31477	5.02380
0.3	0.5	0.2	2.5	0.5	0.3	2	0.3	0.1	6.30575	4.93647	7.67601	6.28907
								0.3	6.36505	4.98895	7.67656	6.28952
								0.6	6.44644	5.06083	7.67738	6.29019

**Table 3:** The values of LNN for various values of physical parameters.

$\phi$	$\gamma _{1}$	$k_{1}$	$F_{r}$	$M_{1}$	$\lambda _{1}$	$R_{*}$	*Ec*	$\gamma _{2}$	$-\left( \mathrm {Re}_{r}\right) ^{-1/2}Nu_{r}$			
									S-W	M-W	S-EG	M-EG
0.0	0.5	0.2	2.5	0.5	0.2	2	0.3	0.3	0.89759	0.89759	1.66046	1.66046
0.3									0.84042	0.82798	1.53777	1.48787
0.6									2.57113	2.77542	4.86408	4.86852
0.3	0.1	0.2	2.5	0.5	0.2	2	0.3	0.3	1.73633	1.72608	3.01042	2.94749
	0.3								1.02022	1.00584	1.83519	1.77882
	0.6								0.79011	0.77828	1.45399	1.40616
0.3	0.5	0.0	2.5	0.5	0.2	2	0.3	0.3	0.80346	0.80600	1.45355	1.41335
		0.2							0.84042	0.82798	1.53777	1.48787
		0.4							0.89675	0.88058	1.63955	1.58974
0.3	0.5	0.2	2.0	0.5	0.2	2	0.3	0.3	0.82857	0.81889	1.51466	1.46863
			2.5						0.84042	0.82798	1.53777	1.48787
			3.0						0.85264	0.83747	1.56093	1.50722
0.3	0.5	0.2	2.5	0.0	0.2	2	0.3	0.3	0.83223	0.82118	1.52155	1.47226
				0.5					0.84042	0.82798	1.53777	1.48787
				1.0					0.86785	0.85278	1.58886	1.53842
0.3	0.5	0.2	2.5	0.5	$\lambda _{1}0.0$	2	0.3	0.3	0.94129	0.91127	1.58115	1.52935
					0.3				0.80987	0.80436	1.51694	1.46812
					0.6				0.76941	0.78849	1.45819	1.41335
0.3	0.5	0.2	2.5	0.5	0.3	$R_{*}0$	0.3	0.3	0.66675	0.65078	1.19081	1.13163
						1			0.74026	0.72974	1.35494	1.30096
						2			0.80987	0.80436	1.51694	1.46812
0.3	0.5	0.2	2.5	0.5	0.3	2	0.0		0.37023	0.37791	0.38638	0.39339
							0.3		0.80987	0.80436	1.51694	1.46812
							0.6		1.91135	1.95652	3.30276	3.22009
0.3	0.5	0.2	2.5	0.5	0.3	2	0.3	0.1	0.28099	0.279376	0.50682	0.49051
								0.3	0.80987	0.804359	1.51694	1.46812
								0.6	1.52963	1.51698	3.02342	2.9261

**Table 4:** Experimental parameters and their level.

	Parameter	Symbol	Level		
			Low $\left( -1\right)$	Medium $\left( 0\right)$	High $\left( 1\right)$
SFC	$\phi$	$A_{1}$	0.1	0.4	0.7
	$F_{r}$	$B_{1}$	1.5	2.0	2.5
	$k_{1}$	$C_{1}$	0.2	0.4	0.6
NN	$\phi$	$A_{2}$	0.1	0.4	0.7
	$R_{*}$	$B_{2}$	0.4	0.8	1.2
	$\gamma _{2}$	$C_{2}$	0.2	0.4	0.6

**Table 5:** Design of experments and response results.

Runs	Coded values			Real values			Response			
	$A_{1}$	$B_{1}$	$C_{1}$	$\phi$	$F_{r}$	$k_{1}$	$\left( \mathrm {Re}_{r}\right) ^{1/2}C_{fr}$			
							S-W	M-W	S-EG	M-EG
1	$-1$	$-1$	$-1$	0.1	1.5	0.2	4.99777	4.26746	5.52335	5.08160
2	1	$-1$	$-1$	0.7	1.5	0.2	0.304264	0.237506	13.0974	11.1701
3	$-1$	1	$-1$	0.1	2.5	0.2	5.99238	5.16796	6.44237	5.92473
4	1	1	$-1$	0.7	2.5	0.2	0.217259	0.346675	14.5054	12.1097
5	$-1$	$-1$	1	0.1	1.5	0.6	7.1343	6.37887	7.62603	7.17184
6	1	$-1$	1	0.7	1.5	0.6	0.0904535	0.491551	41.7064	39.5899
7	$-1$	1	1	0.1	2.5	0.6	8.11852	7.26943	8.54623	8.01598
8	1	1	1	0.7	2.5	0.6	0.0819802	0.0531033	43.1419	40.5550
9	$-1$	0	0	0.1	2.0	0.4	6.57006	5.78109	7.03748	6.55173
10	1	0	0	0.7	2.0	0.4	0.0471869	0.0518682	28.1639	25.8908
11	0	$-1$	0	0.4	1.5	0.4	0.120109	0.087594	9.94356	8.46322
12	0	1	0	0.4	2.5	0.4	0.121533	0.0909836	11.1408	9.37244
13	0	0	$-1$	0.4	2.0	0.2	0.192233	0.255671	7.79098	6.19886
14	0	0	1	0.4	2.0	0.6	0.156307	0.121322	13.2657	11.6068
15	0	0	0	0.4	2.0	0.4	0.111203	0.0860028	10.5421	8.91771
16	0	0	0	0.4	2.0	0.4	0.111203	0.0860028	10.5421	8.91771
17	0	0	0	0.4	2.0	0.4	0.111203	0.0860028	10.5421	8.91771
18	0	0	0	0.4	2.0	0.4	0.111203	0.0860028	10.5421	8.91771
19	0	0	0	0.4	2.0	0.4	0.111203	0.0860028	10.5421	8.91771
20	0	0	0	0.4	2.0	0.4	0.111203	0.0860028	10.5421	8.91771

**Table 6:** Design of experments and response results.

Runs	Coded values			Real values			Response			
	$A_{2}$	$B_{2}$	$C_{2}$	$\phi$	$R_{*}$	$\gamma _{2}$	$-\left( \mathrm {Re}_{r}\right) ^{-1/2}Nu_{r}$			
							S-W	M-W	S-EG	M-EG
1	$-1$	$-1$	$-1$	0.1	0.4	0.2	0.31558	0.30912	0.48793	0.47442
2	1	$-1$	$-1$	0.7	0.4	0.2	4.10256	5.64058	5.96009	6.03155
3	$-1$	1	$-1$	0.1	1.2	0.2	0.38031	0.37592	0.62593	0.61557
4	1	1	$-1$	0.7	1.2	0.2	4.12231	5.68639	6.08449	6.17012
5	$-1$	$-1$	1	0.1	0.4	0.6	0.87231	0.85333	1.45805	1.41765
6	1	$-1$	1	0.7	0.4	0.6	11.2923	15.1317	17.8088	18.0251
7	$-1$	1	1	0.1	1.2	0.6	1.03331	1.01886	1.86839	1.83731
8	1	1	1	0.7	1.2	0.6	11.3372	15.2204	18.1791	18.4378
9	$-1$	0	0	0.1	0.8	0.4	0.66639	0.655553	1.1119	1.08806
10	1	0	0	0.7	0.8	0.4	7.86962	10.6902	12.0203	12.1781
11	0	$-1$	0	0.4	0.4	0.4	1.27867	1.28605	2.37239	2.28721
12	0	1	0	0.4	1.2	0.4	1.35385	1.37051	2.53849	2.46147
13	0	0	$-1$	0.4	0.8	0.2	0.680538	0.687171	1.23029	1.18963
14	0	0	1	0.4	0.8	0.6	1.91227	1.92866	3.67601	3.55473
15	0	0	0	0.4	0.8	0.4	1.31656	1.32859	2.45562	2.37453
16	0	0	0	0.4	0.8	0.4	1.31656	1.32859	2.45562	2.37453
17	0	0	0	0.4	0.8	0.4	1.31656	1.32859	2.45562	2.37453
18	0	0	0	0.4	0.8	0.4	1.31656	1.32859	2.45562	2.37453
19	0	0	0	0.4	0.8	0.4	1.31656	1.32859	2.45562	2.37453
20	0	0	0	0.4	0.8	0.4	1.31656	1.32859	2.45562	2.37453

**Table 7:** Anova analysis for the skin friction coefficient.

Source	DOF	SS	Contribution	Adj. MS	F-value	P-value	
$\left( \mathrm {Re}_{r}\right) ^{1/2}C_{fr}$	SWCNT-Water						
Model	9	160.083	$99.68\%$	17.787	350.1	$2.685\times 10^{-11}$	Significant
Linear	3	104.72	$67.08\%$	34.91	686.98	0	
Square	3	52.165	$32.48\%$	17.39	342.22	0	
Interaction	3	3.198	$1.99\%$	1.066	20.97	0	
Residual Error	10	0.508	$0.32\%$	0.0508	−	−	
Lack of fit	5	0.508	$0.32\%$	0.0508	−	−	
Pure Error	5	0.000	$0.00\%$	0.0000	−	−	
Total	19	160.591	$100\%$	−	−	−	
$\left( \mathrm {Re}_{r}\right) ^{1/2}C_{fr}$	MWCNT-Water						
Model	9	123.358	$99.17\%$	13.71	205.7	$3.771\times 10^{-10}$	Significant
Linear	3	78.487	$63.28\%$	26.16	392.5	0	
Square	3	42.01	$33.87\%$	14.00	210.1	0	
Interaction	3	2.861	$2.31\%$	0.954	14.31	0	
Residual Error	10	0.666	$0.54\%$	0.067	−	−	
Lack of fit	5	0.666	$0.54\%$	0.000	−	−	
Pure Error	5	0.000	$0.00\%$	0.000	−	−	
Total	19	124.024	$100\%$	−	−	−

**Table 8:** Anova analysis for the skin friction coefficient.

Source	DOF	SS	Contribution	Adj. MS	F-value	P-value	
$\left( \mathrm {Re}_{r}\right) ^{1/2}C_{fr}$	SWCNT-Ethylene glycol						
Model	9	2162.69	$98.22\%$	17.787	61.44	$1.435\times 10^{-07}$	Significant
Linear	3	1563.13	$70.99\%$	521.04	133.35	0	
Square	3	247.79	$11.25\%$	82.60	21.12	0	
Interaction	3	351.77	$15.98\%$	117.26	29.98	0	
Residual error	10	39.11	$1.78\%$	3.911	−	−	
Lack of fit	5	39.11	$1.78\%$	7.822	−	−	
Pure error	5	0.000	$0.00\%$	0.000	−	−	
Total	19	2201.8	$100\%$	−	−	−	
$\left( \mathrm {Re}_{r}\right) ^{1/2}C_{fr}$	MWCNT-Ethylene glycol						
Model	9	1989	$98.08\%$	0.013450	56.9	$2.082\times 10^{-07}$	Significant
Linear	3	1376.22	$67.87\%$	458.74	118.11	0	
Square	3	265.82	$13.11\%$	88.61	22.81	0	
Interaction	3	346.96	$17.11\%$	115.65	29.78	0	
Residual error	10	38.84	$1.92\%$	3.884	−	−	
Lack of fit	5	38.84	$1.92\%$	7.768	−	−	
Pure error	5	0.00000	$0.00\%$	0.000	−	−	
Total	19	2027.84	$100\%$	−	−	−

**Table 9:** Anova analysis for the LNN.

Source	DOF	SS	Contribution	Adj. MS	F-value	P-value	
$\left( \mathrm {Re}_{r}\right) ^{-1/2}Nu_{r}$	SWCNT-Water						
Model	9	217.549	$98.70\%$	17.787	84.34	$3.057\times 10^{-08}$	Significant
Linear	3	154.105	$69.92\%$	51.37	179.242	0	
Square	3	41.676	$18.91\%$	13.89	48.474	0	
Interaction	3	21.768	$9.88\%$	7.256	25.319	0	
Residual error	10	2.866	$1.30\%$	0.2866	−	−	
Lack of fit	5	2.866	$1.30\%$	0.5732	−	−	
Pure error	5	0.000	$0.00\%$	0.0000	−	−	
Total	19	220.415	$100\%$	−	−	−	
$\left( \mathrm {Re}_{r}\right) ^{-1/2}Nu_{r}$	MWCNT-Water						
Model	9	417.113	$98.62\%$	13.71	79.3	$4.133\times 10^{-08}$	Significant
Linear	3	287.682	$68.02\%$	95.894	164.09	0	
Square	3	89.653	$21.20\%$	29.884	51.14	0	
Interaction	3	39.778	$9.40\%$	13.259	22.69	0	
Residual error	10	5.844	$1.38\%$	0.067	−	−	
Lack of fit	5	5.844	$1.38\%$	0.000	−	−	
Pure error	5	0.000	$0.00\%$	0.000	−	−	
Total	19	422.957	$100\%$	−	−	−

**Table 10:** Anova analysis for the LNN.

Source	DOF	SS	Contribution	Adj. MS	F-value	P-value	
$\left( \mathrm {Re}_{r}\right) ^{-1/2}Nu_{r}$	SWCNT-Ethylene glycol						
Model	9	522.316	$98.73\%$	58.035	86.55	$2.694\times 10^{-08}$	Significant
Linear	3	378.982	$71.64\%$	126.327	188.391	0	
Square	3	84.272	$15.93\%$	28.091	41.891	0	
Interaction	3	59.062	$11.16\%$	19.687	29.360	0	
Residual error	10	6.706	$1.27\%$	3.911	−	−	
Lack of fit	5	6.706	$1.27\%$	7.822	−	−	
Pure error	5	0.000	$0.00\%$	0.000	−	−	
Total	19	529.022	$100\%$	−	−	−	
$\left( \mathrm {Re}_{r}\right) ^{-1/2}Nu_{r}$	MWCNT-Ethylene glycol						
Model	9	541.614	$98.69\%$	60.179	83.58	$3.197\times 10^{-08}$	Significant
Linear	3	390.082	$71.08\%$	130.027	180.586	0	
Square	3	90.463	$16.48\%$	30.154	41.879	0	
Interaction	3	61.069	$11.13\%$	20.356	28.271	0	
Residual error	10	7.200	$1.31\%$	0.72	−	−	
Lack of fit	5	7.200	$1.31\%$	1.44	−	−	
Pure error	5	0.00000	$0.00\%$	0.000	−	−	
Total	19	548.814	$100\%$	−	−	−

**Table 11:** Estimated regression coefficents for the skin friction coefficient for water.

Term	Coefficients	Std. error	P-value
$\left( \mathrm {Re}_{r}\right) ^{1/2}C_{fr}$	SWCNT-Water		
Constant	0.113267	0.077492	0.174532
*A*	$-3.207189$	0.071282	$7.08\times 10^{-13}$
*B*	0.188478	0.071282	0.024560
*C*	0.387765	0.071282	0.000285
$A^{2}$	3.192259	0.135929	$4.44\times 10^{-10}$
$B^{2}$	0.004457	0.135929	0.974488
$C^{2}$	0.057906	0.135929	0.679131
*AB*	$-0.259289$	0.079696	0.008669
*AC*	$-0.576470$	0.079696	$2.81\times 10^{-05}$
*BC*	0.008518	0.079696	0.917000
	$R^{2}=99.68\%$		$R^{2}-adj=99.4\%$
$\left( \mathrm {Re}_{r}\right) ^{1/2}C_{fr}$	MWCNT-Water		
Constant	0.085376	0.088751	0.358749
*A*	$-2.768411$	0.081639	$1.18\times 10^{-11}$
*B*	0.146517	0.081639	0.102938
*C*	0.403900	0.081639	0.000581
$A^{2}$	2.832044	0.155680	$5.41\times 10^{-09}$
$B^{2}$	0.004853	0.155680	0.975743
$C^{2}$	0.104061	0.155680	0.518989
*AB*	$-0.265042$	0.091275	0.015732
*AC*	$-0.531551$	0.091275	0.000167
*BC*	$-0.069695$	0.091275	0.462761
	$R^{2}=99.46\%$		$R^{2}-adj=98.98\%$

**Table 12:** Estimated regression coefficents for the skin friction coefficient for ethylene glycol.

Term	Coefficients	Std. error	P-value
$\left( \mathrm {Re}_{r}\right) ^{1/2}C_{fr}$	SWCNT-ethylene glycol		
Constant	10.544045	0.679883	$2.54\times 10^{-08}$
*A*	10.543954	0.625401	$1.13\times 10^{-08}$
*B*	0.587996	0.625401	0.369276
*C*	6.692676	0.625401	$8.51\times 10^{-07}$
$A^{2}$	7.053726	1.192594	0.000148
$B^{2}$	$-0.004784$	1.192594	0.996878
$C^{2}$	$-0.018624$	1.192594	0.987848
*AB*	0.125535	0.699220	0.861103
*AC*	6.629870	0.699220	$2.58\times 10^{-06}$
*BC*	0.003585	0.699220	0.996010
	$R^{2}=98.22\%$		$R^{2}-adj=96.62\%$
$\left( \mathrm {Re}_{r}\right) ^{1/2}C_{fr}$	MWCNT-Ethylene glycol		
Constant	8.918313	0.677498	$1.22\times 10^{-07}$
*A*	9.656962	0.623207	$2.56\times 10^{-08}$
*B*	0.450119	0.623207	0.486680
*C*	6.645453	0.623207	$8.80\times 10^{-07}$
$A^{2}$	7.302046	1.188410	0.000109
$B^{2}$	$-0.001389$	1.188410	0.999091
$C^{2}$	$-0.016389$	1.188410	0.989268
*AB*	0.027179	0.696767	0.969653
*AC*	6.585451	0.696767	$2.66\times 10^{-06}$
*BC*	0.003314	0.696767	0.996299
	$R^{2}=98.08\%$		$R^{2}-adj=96.36\%$

**Table 13:** Estimated regression coefficents for the LNN for water.

Term	Coefficients	Std. error	P-value
$\left( \mathrm {Re}_{r}\right) ^{-1/2}Nu_{r}$	SWCNT-water		
Constant	1.32610	0.18404	$2.91\times 10^{-05}$
*A*	3.54561	0.16929	$1.37\times 10^{-09}$
*B*	0.03656	0.16929	0.833
*C*	1.68461	0.16929	$1.66\times 10^{-06}$
$A^{2}$	2.92760	0.32282	$3.86e-06$
$B^{2}$	$-0.02414$	0.32282	0.942
$C^{2}$	$-0.04400$	0.32282	0.894
*AB*	$-0.02013$	0.18927	0.917
*AC*	1.64936	0.18927	$5.53\times 10^{-06}$
*BC*	0.01518	0.18927	0.938
	$R^{2}=98.7\%$		$R^{2}-adj=97.53\%$
$\left( \mathrm {Re}_{r}\right) ^{-1/2}Nu_{r}$	MWCNT-Water		
Constant	1.34639	0.26280	0.000449
*A*	4.91565	0.24174	$1.83\times 10^{-09}$
*B*	0.04513	0.24174	0.855641
*C*	2.14538	0.24174	$4.69\times 10^{-06}$
$A^{2}$	4.29979	0.46099	$3.00\times 10^{-06}$
$B^{2}$	$-0.04480$	0.46099	0.924497
$C^{2}$	$-0.06517$	0.46099	0.890390
*AB*	$-0.01223$	0.27028	0.964806
*AC*	2.22975	0.27028	$8.99\times 10^{-06}$
*BC*	0.01770	0.27028	0.949069
	$R^{2}=98.62\%$		$R^{2}-adj=97.37\%$

**Table 14:** Estimated regression coefficents for the LNN for ethylene glycol.

Term	Coefficients	Std. error	P-value
$\left( \mathrm {Re}_{r}\right) ^{-1/2}Nu_{r}$	SWCNT-Ethylene glycol		
Constant	2.456253	0.281511	$5.46\times 10^{-06}$
*A*	5.450058	0.258952	$1.30\times 10^{-09}$
*B*	0.120914	0.258952	0.651
*C*	2.860162	0.258952	$6.35\times 10^{-07}$
$A^{2}$	4.108897	0.493802	$8.33\times 10^{-06}$
$B^{2}$	$-0.001763$	0.493802	0.997
$C^{2}$	$-0.004053$	0.493802	0.994
*AB*	$-0.006705$	0.289517	0.982
*AC*	2.716343	0.289517	$2.84\times 10^{-06}$
*BC*	0.064780	0.289517	0.827
	$R^{2}=98.73\%$		$R^{2}-adj=97.59\%$
$\left( \mathrm {Re}_{r}\right) ^{-1/2}Nu_{r}$	MWCNT-Ethylene glycol		
Constant	2.375163	0.291710	$1.01\times 10^{-05}$
*A*	5.540966	0.268334	$1.57\times 10^{-09}$
*B*	0.128634	0.268334	0.642
*C*	2.879130	0.268334	$8.31\times 10^{-07}$
$A^{2}$	4.256968	0.511693	$8.34\times 10^{-06}$
$B^{2}$	$-0.001772$	0.511693	0.997
$C^{2}$	$-0.003932$	0.511693	0.994
*AB*	$-0.001193$	0.300007	0.997
*AC*	2.762033	0.300007	$3.37\times 10^{-06}$
*BC*	0.069080	0.300007	0.823
	$R^{2}=98.69\%$		$R^{2}-adj=97.51\%$

**Table 15:** Sensitivity analysis for the SFC when $A=0$.

*B*	*C*	Sensitivity					
		SWCNT-Water			MWCNT-Water		
		$\frac{\partial C_{fr}^{1}}{\partial A}$	$\frac{\partial C_{fr}^{1}}{ \partial B}$	$\frac{\partial C_{fr}^{1}}{\partial C}$	$\frac{\partial C_{fr}^{2}}{\partial A}$	$\frac{\partial C_{fr}^{2}}{\partial B}$	$\frac{ \partial C_{fr}^{2}}{\partial C}$
$-1$	$-1$	0.628569	$-0.205910$	0.263435	$-1.97182$	0.206506	0.265473
	0	0.052099	$-0.197392$	0.379247	$-2.50337$	0.136811	0.473595
	1	$-0.524371$	$-0.188874$	0.495059	$-3.03492$	0.067116	0.681717
0	$-1$	0.36928	$-0.196996$	0.271953	$-2.23686$	0.216212	0.195778
	0	$-0.20719$	$-0.188478$	0.387765	$-2.76841$	0.146517	0.403900
	1	$-0.78366$	$-0.179960$	0.503577	$-3.29996$	0.076822	0.612022
1	$-1$	0.109991	$-0.188082$	0.280471	$-2.50190$	0.225918	0.126083
	0	$-0.466479$	$-0.179564$	0.396283	$-3.03345$	0.156223	0.334205
	1	$-1.04295$	$-0.171046$	0.512095	$-3.56500$	0.086528	0.542327

		SWCNT-Ethylene glycol			MWCNT-Ethylene glycol		
		$\frac{\partial C_{fr}^{3}}{\partial A}$	$\frac{\partial C_{fr}^{3}}{\partial B}$	$\frac{\partial C_{fr}^{3}}{\partial C}$	$\frac{\partial C_{fr}^{4}}{\partial A}$	$\frac{\partial C_{fr}^{4}}{\partial B}$	$\frac{ \partial C_{fr}^{4}}{\partial C}$
$-1$	$-1$	3.78855	0.593979	6.72634	3.04433	0.449583	6.67492
	0	10.4184	0.597564	6.68909	9.62978	0.452897	6.64214
	1	17.0483	0.601149	6.65184	16.2152	0.456211	6.60936
0	$-1$	3.91408	0.584411	6.72992	3.07151	0.446805	6.67823
	0	10.5440	0.587996	6.69268	9.65696	0.450119	6.64545
	1	17.1738	0.591581	6.65543	16.2424	0.453433	6.61267
1	$-1$	4.03962	0.574843	6.73351	3.09869	0.444027	6.68155
	0	10.6695	0.578428	6.69626	9.68414	0.447341	6.64877
	1	17.2994	0.582013	6.65901	16.2696	0.450655	6.61599

**Table 16:** Sensitivity analysis for the LNN when $A=0$.

*B*	*C*	Sensitivity					
		SWCNT-Water			MWCNT-Water		
		$\frac{\partial Nu_{r}^{1}}{\partial A}$	$\frac{\partial Nu_{r}^{1}}{ \partial B}$	$\frac{\partial Nu_{r}^{1}}{\partial C}$	$\frac{\partial Nu_{r}^{2}}{\partial A}$	$\frac{\partial Nu_{r}^{2}}{\partial B}$	$\frac{ \partial Nu_{r}^{2}}{\partial C}$
$-1$	$-1$	1.91638	0.06966	1.75743	2.69813	0.11703	2.25802
	0	3.56574	0.08484	1.66943	4.92788	0.13473	2.12768
	1	5.21510	0.10002	1.58143	7.15763	0.15243	1.99734
0	$-1$	1.89625	0.02138	1.77261	2.68590	0.02743	2.27572
	0	3.54561	0.03656	1.68461	4.91565	0.04513	2.14538
	1	5.19497	0.05174	1.59661	7.14540	0.06283	2.01504
1	$-1$	1.87612	$-0.02690$	1.78779	2.67367	$-0.06217$	2.29342
	0	3.52548	$-0.01172$	1.69979	4.90342	$-0.04447$	2.16308
	1	5.17484	0.00346	1.61179	7.13317	$-0.02677$	2.03274

		SWCNT-Ethylene glycol			MWCNT-Ethylene glycol		
		$\frac{\partial Nu_{r}^{3}}{\partial A}$	$\frac{\partial Nu_{r}^{3}}{ \partial B}$	$\frac{\partial Nu_{r}^{3}}{\partial C}$	$\frac{\partial Nu_{r}^{4}}{\partial A}$	$\frac{\partial Nu_{r}^{4}}{\partial B}$	$\frac{ \partial Nu_{r}^{4}}{\partial C}$
$-1$	$-1$	2.74042	0.059660	2.80349	2.78013	0.063098	2.81791
	0	5.45676	0.124440	2.79538	5.54216	0.132178	2.81005
	1	8.17311	0.189220	2.78728	8.30419	0.201258	2.80219
0	$-1$	2.73372	0.056134	2.86827	2.77893	0.059554	2.88699
	0	5.45006	0.120914	2.86016	5.54097	0.128634	2.87913
	1	8.16640	0.185694	2.85206	8.30300	0.197714	2.87127
1	$-1$	2.72701	0.052608	2.93305	2.77774	0.05601	2.95607
	0	5.44335	0.117388	2.92494	5.53977	0.12509	2.94821
	1	8.15970	0.182168	2.91684	8.30181	0.19417	2.94035

**Table 17:** Comparative values of $f^{\prime \prime }\left( 0\right)$ and $g^{\prime }\left( 0\right)$ for value of $F_{r}=0.2$ when $\gamma _{1}\rightarrow \infty ,$ $\lambda =0.2,$ $S_{1}=0=M_{1}=\phi =\delta _{1}$.

	Present results		Naqvi et al.^46^	
$F_{r}$	$f^{\prime \prime }\left( 0\right)$	$g^{\prime }\left( 0\right)$	$f^{\prime \prime }\left( 0\right)$	$g^{\prime }\left( 0\right)$
	0.43478	$-0.78139$	0.4347813	$-0.7813904$

## Concluding remarks

A numerical investigation on heat transfer improvement corresponds to Darcy–Forchheimer flow of carbon nanotubes along radiative stretched rotating disk using response surface methodology (RSM). The traditional heat transfer liquids such as water, thermal liquids and ethylene glycol, are widely used in various industrial processes involving refrigeration and air conditioning, transportation, solar thermal and microelectronics. Here we us water and ethylene glycol are considered a basic fluid. Main findings are listed below, which offered preliminary guideline for lab-based experimenters in future device of solar-thermal, air-conditioning, refrigeration, transportation and microelectronics:

- The normal $Q--Q$ residual plot presents the best fitted regression model for SFC and LNN for both SWCNT and MWCNT when water and ethylene glycol are taken as base fluids.
- The factors *A*,  *C*,  $A^{2}$ and *AC* corresponds to SWCNT-Ethylene glycol and MWCNT-Ethylene glycol are significant for skin friction coefficient.
- The factors *A*,  *C*,  $A^{2}$ and *AC* corresponds to both SWCNT and MWCNT (for both Water and Ethylene glycol as base fluid) are important for local nusselt number.
- Sensitivity of SFC via SWCNT-water towards the permeability parameter is higher than solid volume fraction for $C=0$ and $C=1.$
- For lower permeability number, the SFC for SWCNT-water seems to have a high sensitivity towards the solid volume fraction instead of permeability and inertia coefficient.
- The sensitivity of LNN for SWCNT-water towards Biot number is approximately equal despite the increment in Biot and radiation number.
- Sensitivity of LNN for MWCNT-water towards the solid volume fraction is higher than the radiation and Biot number for $C=1,0,1.$

## List of symbols

$$\theta$$: Dimensionless temperature
*f*: Dimensionless velocity
$${\check{\rho }}_{n_{f}}$$: Nanofluid’s density
${\check{\mu }}_{n_{f}}$: Nanofluid’s dynamic viscosity
$\rho _{p}$: Density of nanomaterials
$\beta _{nf}$: Is volume expansion coefficient of fluid
$\check{T}$: Temperature of liquid
$\tau$: Heat capacity ratio of nanomaterials by nanofluid
$\sigma$: Electrical conductivity of nanoliquid
$$\check{T}_{\infty }$$: Ambient temperature
$\phi$: Solid volume fraction
$\left( \check{u},\check{v},\check{w}\right)$: Velocity components in $\left( r,\varphi ,z\right)$ directions respectively
$k_{_{CNT}}$: CNTs thermal conductivity,
$k_{_{f}}$: Base fluid’s thermal conductivity
$(\rho c_{p})_{_{CNT}}$: CNTs heat capacity
$$U_{w}$$: Stretching velocity
*g*: Gravity
$$c_{p}$$: Specific heat
$k_{n_{f}}$: Nanofluid’s thermal conductivity
$h_{s}$: Heat transfer coefficient
$$F_{r}$$: Inertia coefficient
$\gamma _{1}$: Casson fluid parameter
$$\Pr$$: Prandtl number
*Ec*: Eckert number
$\Omega$: Constant angular velocity
$h_{f}$: Coefficient of heat transfer
$$\gamma _{2}$$: Biot number
$\lambda$: Mixed convective number
$$R_{*}$$: Radiation parameter
$$S_{1}$$: Suction parameter
$\delta _{1}$: Stretching-strength parameter

## Abbreviations

DOF: Degrees of freedom
SFC: Skin friction coefficient
S-W: SWCNT-Water
M-W: MWCNT-Water
DF: Darcy–Forchheimer
MMS: Modified mean square
EG: Ethylene glycol
LNN: Local Nusselt Number
S-EG: SWCNT-ethylene glycol
M-EG: MWCNT-ethylene glycol
SS: Sum of squares
$${\bar{R}}^{2}$$: Adjusted *R*-Squared
